# Multicenter Performance Evaluation of MALDI-TOF MS for Rapid Detection of Carbapenemase Activity in Enterobacterales: The Future of Networking Data Analysis With Online Software

**DOI:** 10.3389/fmicb.2021.789731

**Published:** 2022-01-27

**Authors:** Eva Gato, Ahalieyah Anantharajah, Manuel J. Arroyo, María José Artacho, Juan de Dios Caballero, Ana Candela, Kateřina Chudějová, Ignacio Pedro Constanso, Cristina Elías, Javier Fernández, Jesús Jiménez, Pilar Lumbreras, Gema Méndez, Xavier Mulet, Patricia Pérez-Palacios, Belén Rodríguez-Sánchez, Rafael Cantón, Jaroslav Hrabák, Luis Mancera, Luis Martínez-Martínez, Antonio Oliver, Álvaro Pascual, Alexia Verroken, Germán Bou, Marina Oviaño

**Affiliations:** ^1^Servicio de Microbiología, Red Española de Investigación en Patología Infecciosa, Instituto de Investigación Biomédica da Coruña, CIBER de Enfermedades Infecciosas (CIBERIFEC), Instituto de Salud Carlos III, Complejo Hospitalario Universitario A Coruña, A Coruña, Spain; ^2^Service de Microbiologie, Cliniques Universitaires Saint-Luc, Brussels, Belgium; ^3^Clover BioSoft, Granada, Spain; ^4^Unidad de Gestión Clínica de Microbiología, Red Española de Investigación en Patología Infecciosa, Hospital Universitario Reina Sofía, CIBER de Enfermedades Infecciosas (CIBERIFEC), Instituto de Salud Carlos III, Córdoba, Spain; ^5^Servicio de Microbiología, Red Española de Investigación en Patología Infecciosa, Hospital Universitario Ramón y Cajal, Instituto Ramón y Cajal de Investigación Sanitaria, CIBER de Enfermedades Infecciosas (CIBERIFEC), Instituto de Salud Carlos III, Madrid, Spain; ^6^Servicio de Microbiología, Hospital General Universitario Gregorio Marañón, Madrid, Spain; ^7^Department of Microbiology, Faculty of Medicine, University Hospital in Pilsen, Charles University, Pilsen, Czechia; ^8^Servicio de Análisis Clínicos, Complejo Hospitalario Universitario A Coruña, A Coruña, Spain; ^9^Instituto Maimónides de Investigación Biomédica de Córdoba, Córdoba, Spain; ^10^Servicio de Microbiología, Hospital Universitario Central de Asturias, Instituto de Investigación Sanitaria del Principado de Asturias, Oviedo, Spain; ^11^Servicio de Microbiología, Hospital Universitario Son Espases, Red Española de Investigación en Patología Infecciosa, CIBER de Enfermedades Infecciosas (CIBERIFEC), Instituto de Salud Carlos III, Palma, Spain; ^12^Unidad Clínica de Enfermedades Infecciosas y Microbiología Clínica, CSIC, Red Española de Investigación en Patología Infecciosa, Hospital Universitario Virgen Macarena, Instituto de Biomedicina de Sevilla, CIBER de Enfermedades Infecciosas (CIBERIFEC), Instituto de Salud Carlos III, Universidad de Sevilla, Seville, Spain; ^13^Departamento de Química Agrícola, Edafología y Microbiología, Universidad de Córdoba, Córdoba, Spain

**Keywords:** MALDI-TOF MS, carbapenemases enzymes, resistance detection, imipenem, clinical microbiology

## Abstract

In this study, we evaluate the performance of matrix-assisted laser desorption/ionization time-of-flight mass spectrometry (MALDI-TOF MS) for rapid detection of carbapenemase activity in Enterobacterales in clinical microbiology laboratories during a multicenter networking validation study. The study was divided into three different stages: “software design,” “intercenter evaluation,” and “clinical validation.” First, a standardized procedure with an online software for data analysis was designed. Carbapenem resistance was detected by measuring imipenem hydrolysis and the results were automatically interpreted using the Clover MS data analysis software (Clover BioSoft, Spain). Second, a series of 74 genotypically characterized Enterobacterales (46 carbapenemase-producers and 28 non carbapenemase-producers) were analyzed in 8 international centers to ensure the reproducibility of the method. Finally, the methodology was evaluated independently in all centers during a 2-month period and results were compared with the reference standard for carbapenemase detection used in each center. The overall agreement rate relative to the reference method for carbapenemase resistance detection in clinical samples was 92.5%. The sensitivity was 93.9% and the specificity, 100%. Results were obtained within 60 min and accuracy ranged from 83.3 to 100% among the different centers. Further, our results demonstrate that MALDI-TOF MS is an outstanding tool for rapid detection of carbapenemase activity in Enterobacterales in clinical microbiology laboratories. The use of a simple in-house procedure with online software allows routine screening of carbapenemases in diagnostics, thereby facilitating early and appropriate antimicrobial therapy.

## Introduction

Carbapenemase-producing Enterobacterales (CPE) have been recognized by a number of national and international health organizations to represent a major threat to global health (Rodríguez-Baño et al., 2018; U.S. Centers for Disease Control and Prevention, 2019; European Center for Disease Prevention and Control, 2020). Therefore, the search for new, rapid and effective tools for detecting these bacteria in microbiology laboratories is essential to enable early targeted antibiotic therapy. Numerous phenotypic, lateral flow and DNA-based methods have been used in the laboratory with the aim of detecting CPE (Hrabák et al., 2014). The results of culture-based methods are generally available within 24 h of isolation of the bacteria from clinical samples, whereas more expensive genotypic test results are available within hours. Moreover, DNA-based methods can only detect a predefined range of carbapenem-encoding genes, the presence of which does not guarantee expression (Decousser et al., 2015; Dortet and Naas, 2017; Oueslati et al., 2019).

Since the introduction of MALDI-TOF in clinical microbiology laboratories, approaches to detect carbapenemases have been made by using MALDI-TOF MS-based measurement of the hydrolysis of different antibiotics (Clark et al., 2013; Papagiannitsis et al., 2015; Oviaño and Bou, 2018; Oviaño et al., 2019). However, several in-house MALDI-TOF MS assays that use different antibiotic combinations, buffers and variable incubation times, ranging from 15 min to 4 h (Burckhardt and Zimmermann, 2011; Hrabák et al., 2011; Lasserre et al., 2015; Monteferrante et al., 2016; Carvalhaes et al., 2018), have been described. In its guidelines for detection of resistance mechanisms, version 2.0 of July 2017, the EUCAST committee commented on the detection of carbapenem hydrolysis by MALDI-TOF MS. Although the method was recommended for detection of carbapenemases in Enterobacterales, the document highlighted the lack of evaluation of the procedure in a multicenter study design or in studies involving multiple individual centers as a limitation to its use. Hence, standardization of the methodology is required before universal application of MALDI-TOF for the detection of carbapenemase activity based on hydrolysis of a carbapenem antibiotic.

In line with the standardization and automation of the process, the MBT STAR^®^-Carba IVD kit (Bruker Daltonik, Germany) and associated MBT STAR^®^-BL IVD software have become available for a fully developed workflow, with a benchmark carbapenem antibiotic, in Bruker equipment (Anantharajah et al., 2019; Cordovana et al., 2020). However, as a result of the increasing need to share data among different laboratories we have developed an online software for mass spectra data analysis that is universally compatible with any mass spectra format derived from any MALDI-TOF commercial brand, so it is independent from suppliers other than the commercial companies who distribute MALDI-TOF MS equipment.

The aim of this study was to conduct a multicenter evaluation of the accuracy and applicability of MALDI-TOF MS for rapid detection of CPE based on the hydrolysis of imipenem with a standardized in-house procedure and subsequent automated interpretation of the results with online mass spectra data analysis software.

## Materials and Methods

### Study Design

The study was divided into three different stages. The first part, i.e., “software design,” was carried out to implement online mass spectra data analysis software. To this end, the imipenem hydrolysis assay conditions were the same as those established by Oviaño et al. (2019), using a collection of 74 genotypically characterized CPE and data analysis with the Clover MS data analysis software (Clover BioSoft, Spain)^1^. The results were compared with those obtained with the MBT STAR^®^-Carba IVD Kit (Bruker Daltonik) and STAR^®^-BL IVD software. The principle of the assay is that imipenem is inactivated due to hydrolysis of the β-lactam ring by bacteria expressing carbapenemase-hydrolyzing enzymes. The hydrolysis reaction modifies the structure of the antibiotic, so disappearance in the native mass peaks (300 and 489 m/z) can be detected by MALDI-TOF. Thus, a positive hydrolysis reaction denotes the presence of a carbapenemase in bacteria. This first part of the study process was accomplished by the Complejo Hospitalario Universitario A Coruña (AC), A Coruña, considered the reference center.

The second part of the study, i.e., “intercenter evaluation,” was designed to standardize the methodology and evaluate the reproducibility among the different centers participating in the survey with the proposed workflow. The same isolates used in the first part of the study were analyzed in the eight participating centers: the Hospital General Universitario Gregorio Marañón (GM), Madrid; the Hospital Universitario Ramón y Cajal (RC), Madrid; the Hospital Universitario Reina Sofía (RS), Córdoba; the Hospital Universitario Virgen Macarena (VM), Seville; the Hospital Universitario Son Espases (SE), Palma; the Hospital Universitario Central de Asturias (CA), Oviedo; the University Hospital Plzeň (PZ), Plzeň; and the Cliniques Universitaires Saint-Luc (SL), Brussels. All reagents were provided by the reference center and solutions were prepared in all centers according to instructions. So, the centers were given a precise protocol with the standardized procedure and an educational video for software management (Supplementary Data Sheet 1 and Supplementary Video 1). Technical support regarding the laboratory procedure and general attention together with software management were available during the study. The results of the MALDI-TOF assay were compared with those obtained by molecular characterization of the isolates.

The last part of the study, i.e., “clinical validation,” was designed to evaluate the proposed methodology and data analysis in real clinical settings. During a 2 months period, all isolates suspected by EUCAST screening criteria (i.e., with a meropenem or ertapenem MIC > 0.125 mg/L) to be carrying a carbapenemase enzyme (maximum 20) were evaluated in parallel in the participating centers using the MALDI-TOF MS assay based on imipenem hydrolysis. The results obtained were compared with those yielded by the reference method used in each clinical laboratory for CPE detection.

### Bacterial Isolates

A representative collection of 76 non-duplicated clinical isolates (retrospectively obtained from our own collection of isolates belonging to AC) was used in the first and second parts of the study. These included 74 isolates (46 CPE and 28 non-CPE) and 2 control strains. The control strains used were a *E. coli* ATCC 25922 (negative control) and a PCR-confirmed VIM-producing *Citrobacter freundii* (positive control). The 74 test species included in the study were 39 *Klebsiella pneumoniae*, 26 *Escherichia coli*, 8 *Enterobacter cloacae* complex, and 1 *Klebsiella oxytoca*. The isolates were characterized by PCR and sequencing (Oteo et al., 2013, 2016; Dortet et al., 2016; Oviaño et al., 2017). The collection included 46 CPE isolates, being 10 *bla*_*KPC*_, 3 *bla*_*IMP*_, 5 *bla*_*NDM*_, 10 *bla*_*VIM*_ and 18 *bla*_*OXA–48–like*_, and 28 non-CPE isolates: 1 *bla*_*CIT*_, 1 *bla*_*SHV*_, 2 *bla*_*CMY*_, 3 *bla*_*FOX*_, 1 *bla*_*K–1*_, 14 *bla*_*CTX–M*_, and 6 fully susceptible isolates (see Supplementary Table 1 for further details). All non-CPE isolates have carbapenems MIC below the cut-off for screening according to EUCAST criteria, excepting two isolates, one *E. coli* and 1 *K. pneumoniae*. Both were tested by PCR and sequencing and no carbapenemase enzyme was found.

In the final part of the study, each center prospectively evaluated (following EUCAST screening guidelines) suspected CPE isolates that emerged in routine testing in clinical microbiology laboratories, i.e., the number of isolates that emerged during the 2 months period or up to 20 isolates. One isolate per patient included. The isolates were derived from different clinical samples. Results were compared with those obtained with the reference method used in each laboratory.

### MALDI-TOF MS Acquisition

Bacterial isolates were stored at −80°C, in a small vial containing glass cryopearls (Deltalab, Barcelona, Spain). On day 1, isolates were thawed on a blood agar plate by removing one of the pearls from the tube with a sterile loop and rolling it on the plate. After incubation for 18 h, the isolates were subcultured for another 18 h on a blood agar plate for analysis under standard conditions. The samples were incubated in an aerobic atmosphere at 37°C. Briefly, bacteria filling a 1 μl inoculation loop were suspended in 50 μl of solution (10 mM NH_4_CO_3_, 10 μg/ml ZnCl_2_, 0.001% SDS; pH 8) containing 0.5 mg/mL of imipenem (Sigma-Aldrich, Germany) and incubated for 30 min at 37°C with slow agitation (300 rpm) (Oviaño et al., 2019). The samples were then centrifuged at 14,000 rpm, and the supernatant was applied to a MALDI-TOF MS target plate. Each sample was spotted on the plate in duplicate. Once dried, 1 μl of matrix [Matrix IVD HCCA-portioned (Bruker Daltonik) spiked with 1 ppm/μl of reserpine (Sigma-Aldrich, Germany)] was applied to each spot. All runs were performed in the presence of the positive and negative controls treated in the same way as the samples. Because of the instability of imipenem, the antibiotic solution must be prepared just before use, or freezed at −20°C for 1 week or at −70°C for a month. The negative control is useful to evaluate spontaneous hydrolysis of the antibiotic.

The configuration of the equipment was the Research Use Only version as the IVD version does not allow access to the modification of parameters in the FlexControl software. Appropriate calibration was conducted before each run using a mixture of bradykinin [1-5] and [1-7] at 35 μM (Sigma-Aldrich). The mixture was spotted and once dried 1 μl of matrix was added on top of each spot. The spectra were acquired after the mixture was dried.

The mass spectra were obtained using a MALDI Biotyper^®^ Smart (Bruker Daltonik) system, with Flex Control 3.4 software. Three methods were created for spectral acquisition: MBT_ATB.par, MBT_ATB_AutoX.axe and MBT_ATB.mcl. The operational mass range was between 100 and 1,000 m/z in the linear positive mode. The mass peaks were acquired in 40 shot steps to produce 240 satisfactory shots, and the resolution of the mass peaks selected was higher than 300. The movement of the laser in the spot followed a large spiral.

The parameters considered in the calibration are listed in Table 1.

**TABLE 1:** MALDI-TOF MS calibration parameters.

Name	m/z	Resolution	Intensity
[HCCA+H]^+^	190.050	>300	>3000
[2*HCCA* + *H*]^+^	379.092	>500	>3000
[*Bradykinin*(1−5)]^+^	573.314	>700	>2000
[*Reserpine*]^+^	607.680	>800	>3000
[*Bradykinin*(1−7)]^+^	757.400	>1000	>3000

*The mass peaks considered for calibration, including the exact masses, resolutions and intensities.*

### MALDI-TOF MS Data Analysis

In the first part of the study, i.e., “software design,” the results were analyzed in parallel, with our in-house imipenem hydrolysis procedure and Clover MS data analysis software (Clover BioSoft, Spain) and compared with those obtained with the MBT STAR^®^-Carba IVD Kit (Bruker Daltonik) and the STAR^®^-BL IVD software.

**FIGURE 1:**
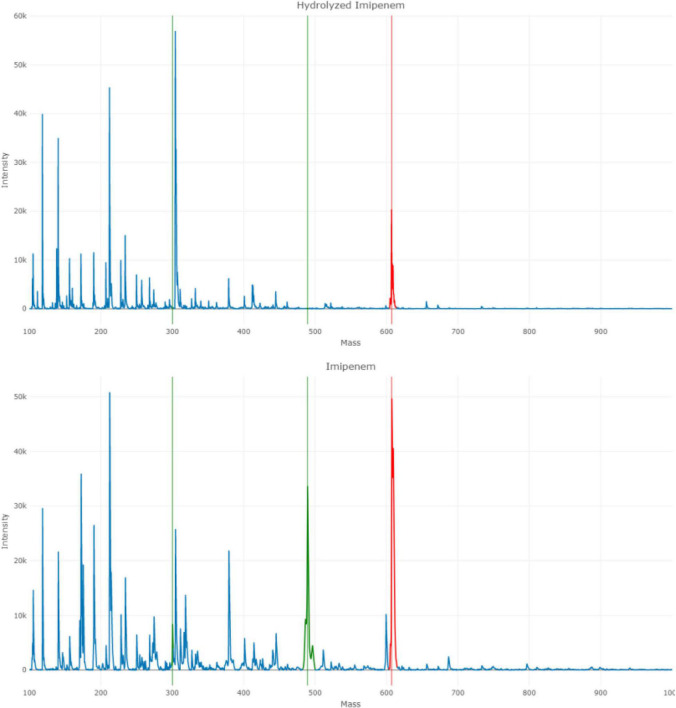
Imipenem spectra. The hydrolyzed imipenem spectrum, in the first picture, represents no visible mass peaks for imipenem and only the internal standard, reserpine, is observed at 607 m/z. The later spectrum corresponds to imipenem, with the mass peaks at 300 and 489 m/z and the internal standard at 607 m/z.

Instructions for using Clover MS data analysis software (Clover BioSoft, Spain) in the Carbapenem Hydrolysis Detection Analysis module are provided in Supplementary Data Sheet 1 and Supplementary Video 1. The software automatically performs the baseline subtraction and subsequently calculates the ratio of hydrolysis (RH) of imipenem, based on the ratios of the mass peaks of imipenem (300 m/z) and imipenem complexed with the matrix (489 m/z) and the internal standard, i.e., reserpine (607 m/z) (Figure 1). The mass peak at 300 m/z is usually less intense than the 489 m/z as imipenem tends to stabilize forming an adduct with the matriz (Oviaño and Bou, 2018). The results were normalized according to the controls. Results are displayed in a .pdf report (Supplementary Data Sheet 2).

## Results

### Software Design

In the STAR^®^-BL IVD software, (a) the cut-off values used were those recommended by the manufacturer. In the Clover MS data analysis software (Clover BioSoft), the RH was calculated in two different ways: (b) by using the intensity of the imipenem mass peaks and (c) by using the area under the curve (AUC) of the peaks. A receiver operating characteristic (ROC) curve was calculated for the three methods, yielding AUC values of (a) 0.977 (0.939–1), (b) 0.994 (0.985–1), and (c) 0.990 (0.976–1), with a 95% confidence interval. As the best results were yielded by the analysis that integrated the mass peaks using intensity (b), the spectra were then treated in this way in the rest of the study. The cut-off values for the in-house developed assay were determined using the ROC curve and the Youden index. The cut-off value established for positivity in the imipenem hydrolysis assay was RH values equal to or above 0.5. Values below 0.2 were considered to indicate negative hydrolysis. A gray or intermediate zone was established between 0.2 and 0.5, which required further testing, including prolonged incubation times, or confirmation by other techniques. The negative and positive controls were each analyzed 20 times in the same run to ensure repeatability. A minimum delta RH value between the positive and negative control of three times the value of the standard deviation was established. The value was established in 0.6. Thus, the positive control should have values ≥0.5, the negative control values <0.2 and the minimum delta RH value should be 0.6.

For comparing which technique yielded best results and if these differences were statistically significant we analyzed the ROC curve. As the AUC for the STAR^®^-BL IVD software (a) was 0.977 and this value is out of the 95% confidence interval of the AUC of our developed software (b), (0.985–1) we can conclude that the performance of our developed Clover MS data analysis software (Clover BioSoft) is significantly better when using the intensity of the mass peaks.

Continuous variables are presented as mean (95% confidence interval). According to the cut-off values recommended by the manufacturers, 95.06% of the isolates were correctly classified in (a) the STAR^®^-BL IVD software, with 92.00% (79.89–97.41) sensitivity and 100% (86.27–99.71) specificity, whereas using (b) the Clover MS data analysis software (Clover BioSoft), 96.30% of the isolates were correctly classified, with 94.00% (82.46–98.44) sensitivity and 100% (86.27–99.71) specificity. The correlation between the two different procedures and data analysis was calculated with the Spearman correlation coefficient, yielding a ρ value of 0.971.

### Intercenter Evaluation

In the second part of the study, the same isolates used in the first part were analyzed by the eight international centers participating in the study (Supplementary Table 2). Two isolates, i.e., OXA-244-producing *K. pneumoniae* and OXA-232-producing *E. coli* although being classified as CPE according to literature, both enzymes have a low carbapenemase activity, even lower than OXA-48 enzyme (Potron et al., 2013; Hoyos-Mallecot et al., 2017; Pitout et al., 2019). Therefore, data on these isolates were not included in the statistical, as were considered outliers. However, the results were consistent with a low level of enzymatic activity. Among the nine centers, one center provided an intermediate result for the OXA-244-producing *K. pneumoniae*, and three centers provided a negative hydrolysis result. In the case of the OXA-232-producing *E. coli*, one center provided an intermediate result and five centers provided negative results for imipenem hydrolysis.

Once all of the results were obtained, the ROC curve was recalculated with the data obtained in our center (CHUAC) plus the 8 participating centers, to strengthen the statistical power of the analysis. For statistical calculations, 1296 spectra were analyzed from duplicate measurements of the 72 isolates included in the study and measured in the nine centers. The cut-off value for positivity was finally established for RH values ≥0.4; negative hydrolysis values yielded RH < 0.2, and the gray zone, with intermediate values, was established for values of RH between 0.2 and 0.4. Thus, only the positivity cut-off changed slightly. The value of the positive control was adjusted to the new value. The overall sensitivity of the procedure was 95.5% (92.9–97.1), with 100% (98.5–100) specificity. The positive predictive value was 100% (99.0–100) and the negative value, 93.3% (89.8–95.7).

For statistical calculations, full agreement was considered when the MALDI-TOF assay provided an imipenem positive hydrolysis result for CPE or a negative hydrolysis result for non-CPE isolates. Three types of errors were also considered. Errors were considered minor when the result of the MALDI-TOF assay was in the intermediate category and the isolate was either CPE or non-CPE. Errors were considered major when the result of the MALDI-TOF assay was positive for imipenem hydrolysis for a non-CPE isolate and very major errors were considered when a negative hydrolysis result was obtained by MALDI-TOF for a CPE. The overall agreement rate was 94.9% (92.9–96.4); with minor errors representing 3.5% (2.4–5.3) and very major errors 1.5% (0.8–2.8) of the total. No major errors were found. The detailed results for each center are shown in Table 2. The best results were obtained in GM and in PZ, with 98.6% agreement, and the worst results were obtained in CA, with 88.9% agreement. Minor errors and very major errors ranged between, respectively, 1.4 and 8.3% and 1.4 and 5.6%. Regarding exclusively carbapenemase-producing isolates, the overall agreement rate was 95.5% (92.9–97.1, with minor errors representing 2.0% (1.0–3.9) and very major errors 2.5% (1.4–4.6) of the total.

**TABLE 2:** Agreement rates for each center in the intercenter evaluation.

Center^1^	Agreement rate^2^ (*n* = 76)	Minor errors^3^	Very major errors^4^
GM	98.6%	1.4%	0%
RC	95.8%	1.4%	2.8%
RS	94.4%	4.2%	1.4%
VM	90.3%	8.3%	1.4%
SE	90.3%	8.3%	1.4%
CA	88.9%	8.3%	2.8%
AC	93.1%	6.9%	0%
PZ	98.6%	1.4%	0%
SL	91.7%	2.8%	5.6%
Total	94.9%	3.5%	1.5%

*^1^The eight participating centers were Hospital General Universitario Gregorio Marañón (GM), Madrid; Hospital Universitario Ramón y Cajal (RC), Madrid; Hospital Universitario Reina Sofía (RS), Córdoba; Hospital Universitario Virgen Macarena (VM), Seville; Hospital Universitario Son Espases (SE), Palma; Hospital Universitario Central de Asturias (CA), Oviedo; University Hospital Plzeň (PZ), Plzeň and Cliniques Universitaires Saint-Luc (SL), Brussels, along with the reference center Complejo Hospitalario Universitario A Coruña (AC), A Coruña.*

*^2^Full agreement was considered when the MALDI-TOF assay provided an imipenem positive hydrolysis result for CPE or a negative hydrolysis result for non CPE.*

*^3^Minor errors were considered when the result of the MALDI-TOF assay was in the intermediate category and the isolate was either CPE or non CPE.*

*^4^Very major errors were considered when the result of the MALDI-TOF assay was negative for imipenem hydrolysis in CPE. No major errors were found in the study.*

The rates of agreement regarding the species are shown in Table 3. Values range from 91.6% (87.2–94.5) for *E. coli* to 100.0% (70.1–100.0) for *K. oxytoca.* The rates of agreement in relation to the resistance mechanisms are shown in Table 4. The rates of agreement ranged from 87.8% for VIM to 100% for K1. Regarding the accuracy of the MALDI-TOF method for detection of carbapenemases, the best classification rates were yielded for KPC-producing bacteria, with 98.9% of agreement relative to molecular methods.

**TABLE 3:** Agreement rates for different species in the intercenter evaluation for all centers participating in the study.

Species	Diagnosis	*N* ^1^	%^2^	CI 95%^3^
*K. pneumoniae*	Agreement rate	329	96.2%	93.6–97.8%
	Minor errors	9	2.6%	1.4–4.9%
	Very major errors	4	1.2%	0.5–3.0%
*E. coli*	Agreement rate	206	91.6%	87.2–94.5%
	Minor errors	13	5.8%	3.4–9.6%
	Very major errors	6	2.7%	1.2–5.7%
*E. cloacae*	Agreement rate	71	98.6%	92.5–99.8%
	Minor errors	1	1.4%	0.2–7.5%
	Very major errors	0	0.0%	0.0–5.1%
*K. oxytoca*	Agreement rate	9	100.0%	70.1–100.0%
	Minor errors	0	0.0%	0.0–29.9%
	Very major errors	0	0.0%	0.0–29.9%

*^1^Number of isolates included in each category.*

*^2^Percentage of isolates from each species that fit in each diagnosis category.*

*^3^The calculations are performed for a 95% confidence interval (CI).*

**TABLE 4:** Agreement rates regarding the resistance mechanism obtained in the intercenter evaluation for all centers participating in the study.

Resistance mechanism	Diagnosis	*N*	%
Susceptible	Agreement rate	51	94.40%
	Minor errors	3	5.60%
	Very major errors	0	0.00%
CIT	Agreement rate	8	88.90%
	Minor errors	1	11.10%
	Very major errors	0	0.00%
SHV	Agreement rate	8	88.90%
	Minor errors	1	11.10%
	Very major errors	0	0.00%
CMY-2	Agreement rate	17	94.40%
	Minor errors	1	5.60%
	Very major errors	0	0.00%
FOX	Agreement rate	24	88.90%
	Minor errors	3	11.10%
	Very major errors	0	0.00%
K-1	Agreement rate	9	100.00%
	Minor errors	0	0.00%
	Very major errors	0	0.00%
CTX-M	Agreement rate	120	95.20%
	Minor errors	6	4.80%
	Very major errors	0	0.00%
KPC	Agreement rate	89	98.90%
	Minor errors	0	0.00%
	Very major errors	1	1.10%
IMP	Agreement rate	26	96.30%
	Minor errors	1	3.70%
	Very major errors	0	0.00%
NDM	Agreement rate	43	95.60%
	Minor errors	0	0.00%
	Very major errors	2	4.40%
VIM	Agreement rate	79	87.80%
	Minor errors	4	4.40%
	Very major errors	7	7.80%
OXA-48-like	Agreement rate	141	97.90%
	Minor errors	3	2.10%
	Very major errors	0	0.00%

Two isolates yielded most of the errors in the “intercenter evaluation,” both are VIM-producing *E. coli.* The first one yielded minor and very major errors in 3 and in 1 out of the 9 centers, respectively. The second one yielded very major errors in 5 out of the 9 centers.

### Clinical Validation

The participating centers GM, RC, RS, PZ, and SL analyzed 20 isolates in the 2-month period; CA analyzed 19 isolates, SE analyzed 18 isolates and VM analyzed 10 isolates (Supplementary Table 3). The 147 isolates included were obtained from different clinical samples, urine (54), colonization samples (51), blood (16), wounds (8), respiratory samples (7), abscesses (5), catheter (4), and biopsies (2). Regarding the reference methods for detection of resistance, GM, CA, and SL used the O.K.N.V immunochromatographic test from CORIS BioConcept (Belgium); RC, PZ, RS, and SE used characterization by PCR and VM by WGS. The species most frequently detected were *K. pneumoniae* (67), followed at some distance by *E. cloacae* (Brackmann et al., 2020) and *E. coli* (Pitout et al., 2019). The carbapenemase enzyme most frequently detected was OXA-48-like (69), followed by VIM (31), KPC (21), NDM (12), and IMP (5). The distribution of the carbapenemase enzymes among the different centers is shown in Table 5.

**TABLE 5:** Distribution of resistance mechanism in relation to the centers in the clinical validation.

Center^1^ (*N*^2^)	Resistance mechanism					
	OXA	VIM	KPC	NDM	IMP	Non-carbapenem producers
GM (20)	20	0	0	0	0	0
RC (20)	9	7	4	0	0	0
RS (20)	6	4	6	0	4	0
VM (10)	5	2	2	0	1	0
SE (18)	1	10	0	0	0	7
CA (19)	16	2	0	0	0	1
PZ (20)	5	4	6	5	0	0
SL (20)	7	2	3	7	0	1
Total (147)	69	31	21	12	5	9

*^1^Hospital General Universitario Gregorio Marañón (GM), Madrid; Hospital Universitario Ramón y Cajal (RC), Madrid; Hospital Universitario Reina Sofía (RS), Córdoba; Hospital Universitario Virgen Macarena (VM), Seville; Hospital Son Espases (SE), Palma; Hospital Universitario Central de Asturias (CA), Oviedo; Plzeň University Hospital (PZ), Plzeň and Cliniques Universitaires Saint-Luc (SL), Brussels.*

*^2^Number of strains used for clinical validation in each center.*

For statistical calculations, a total of 294 spectra were analyzed for duplicate measurements of 147 isolates. The overall agreement rate relative to the reference method for resistance detection was 92.5% (87.1–95.8), with 4.1% minor errors (1.9–8.6) and 3.4% very major errors (1.5–7.7) (Table 6). Thus, the sensitivity of the MALDI-TOF assay for detecting CPE was 93.9% (88.8–96.7). As no major errors were also found in the clinical validation stage the specificity of the methodology is 100% (70.1–100.0). The best results were obtained in VM and in PZ, with 100% of agreement and the worst results were obtained in SE, with 83.3% agreement. Minor and very major errors ranged between 0 and 10.0% and between 0 and 10%, respectively. Regarding the detection of the different carbapenemases enzymes, the agreements rates ranged from 88.4% for an OXA-48-like-producing isolate to 100% for KPC, NDM, and IMP. Minor and very major errors ranged between 0% (for VIM, KPC, NDM, and IMP) and 5.8% (for OXA-48-like).

**TABLE 6:** greement rates for the different centers obtained in the clinical validation stage.

Center (*N*)^1^	Number of CPE^2^	Reference method^3^	Diagnosis		
			Agreement rate	Minor error	Very major error
GM (20)	19	IC	85.0%	5.0%	10.0%
RC (20)	20	PCR	95.0%	5.0%	0.0%
RS (20)	20	PCR	90.0%	10.0%	0.0%
VM (10)	10	WGS	100.0%	0.0%	0.0%
SE (18)	11	PCR	83.3%	5.6%	10.0%
CA (19)	19	IC	94.7%	5.3%	0.0%
PZ (20)	20	PCR	100.0%	0.0%	0.0%
SL (20)	19	IC	95.0%	0.0%	5.0%
Total (147)	138		92.5%	4.1%	3.4%

*^1^Number of strains used for clinical validation in each center.*

*^2^Number of CPE confirmed among all the isolates suspected of having a carbapenemase enzyme by EUCAST screening criteria.*

*^3^The reference method used in each center for comparison with the results obtained with MALDI-TOF is described here. IC stands for the immunochromatographic test from CORIS BioConcept (Belgium), the rest of techniques were molecular based methods, like PCR and WGS.*

## Discussion

MALDI-TOF MS is a simple, rapid procedure for detecting antimicrobial resistance that combines the universal advantages of phenotypic assays with the rapidity and accuracy of molecular assays. Different approaches have been developed for antimicrobial resistance detection by MALDI-TOF, including the quantitative growth assay (Idelevich et al., 2018) and the peak profiling methodology (Josten et al., 2014; Lau et al., 2014; Cordovana et al., 2018; Brackmann et al., 2020; Gato et al., 2021). However, none of these have been recommended by the EUCAST committee. To our knowledge, this is the first multicenter study evaluating the performance of MALDI-TOF MS for detecting carbapenemase activity in Enterobacterales in clinical microbiology laboratories.

At the “software design” stage, we realized that our in-house procedure with data analysis in the Clover MS data analysis software (Clover BioSoft, Spain) yielded stronger agreement relative to the results obtained with the MBT STAR^®^-Carba IVD Kit (Bruker Daltonik) in the STAR^®^-BL IVD software (96.30% versus 92.00%). Our procedure also provides several other advantages. First, it is commercially independent from the manufacturer of the MALDI-TOF instrument, so that different spectra formats are compatible. In this study, all collaborators used Bruker Daltonik equipment; however, the data analysis can also be performed from mass spectra originated with bioMérieux mass spectrometer devices (Marcy-l’Étoile, France) an others, without the need for further modifications (data not shown). The software automatically converts any mass spectra format in a common format used for interpretation of spectra without any previous knowledge from the user. This is a particularly useful feature, as multiple instrument platforms have emerged since the universal integration of MALDI-TOF in microbiology laboratories. Furthermore, the software is available online and the information generated can thus be accessed anywhere at any time. This is a huge advantage for clinical use, as MALDI-TOF instruments are used for high-throughput screening in microbiology laboratories for identification purposes, so the online application allows access from any computer in the lab, releasing the MALDI-TOF computer for identification and delaying the resistance analysis to any suitable moment. The online application also enables networking with colleagues in different centers, so that information can be shared and advice can even be requested from reference centers, as it is currently very common for hospitals to unify forces and work together as bigger working areas that share patients and information. Besides, the software includes the database of spectra introduced and saved by the user, that can be shared upon permission with any other user of the software. In the “intercenter evaluation” results obtained by the different centers were compared with the molecular characterization of the isolates. In the “clinical validation” results were not compared to a unique standard, but to different and independent validated techniques used in real clinical situations for carbapenemase detection in Enterobacterales, facilitating the user familiarization with the developed methodology In the “intercenter evaluation,” we have demonstrated that the procedure developed is highly reproducible, as agreements rates above 90% were reached in all centers, except one, with a range of values across centers of less than 10%. The inexperience of the user plus a non-fully adjusted equipment by an experienced technician could be the reasons of the lower rates. Moreover, in the “clinical validation” stage, the levels of agreement relative to the reference techniques applied in each laboratory were very similar to those obtained in the “intercenter evaluation,” i.e., 92.5% versus 94.9%, thus validating the technique for use in clinical practice. Minor errors did not exceed 5% and very major errors were less than 3.5% in the real clinical setting, thus preventing further unnecessary testing of isolates.

In the “intercenter evaluation,” study of the rates of agreement regarding the species did not indicate any significant differences, with a very similar level of accuracy for different bacteria. Regarding the “clinical validation” stage, no statistical analysis was possible, because of the small number of isolates of each bacterial species.

Regarding the agreement rates in the “clinical validation” stage for the different carbapenemase enzymes, detection of OXA-48-like-producing isolates was slightly poorer than for the other CPE isolates, i.e., 88.4% versus 100%. However, in the “intercenter evaluation,” detection of VIM-producing isolates was poorest, detected at a rate of 87.8%, with OXA-48-like-producing isolates detected with an accuracy rate of 97.9%. Possible reasons for these differences include the presence of different copies of the resistance plasmid and the isolates belonging to different, epidemiologically unrelated sequence types expressing different degrees of susceptibility to carbapenems. However, more extensive evaluation should be performed in order to establish the reasons for these differences.

Neither in the “intercenter evaluation” nor in the “clinical validation” stage, major errors were produced, thus preventing false-positive detection of AmpC or ESBL-producers, errors commonly found in phenotypic methods of carbapenemase detection (Hrabák et al., 2014; European Center for Disease Prevention and Control, 2020).

Regarding the results bracket among the different centers, 88.9–198.6% in the “intercenter evaluation and 83.3–100% in the “clinical validation” stages, the variance is higher in the second part. This can be attributable to the biological differences among isolates and also in the number of CPE confirmed. Not all suspected isolates of having a carbapenemase by EUCAST screening criteria, were confirmed by the reference method used by the respective laboratory. For example, the center having the lowest accuracy data, SE, had only 11 CPE, so errors have higher impact in the final accuracy percentage, than for example in RC, PZ, or SL with 20 CPE analyzed. As only one center used as reference method WGS, false positive results obtained by MALDI-TOF could be wrongly attributable, really corresponding to true carbapenemase isolates rarely detected as IMI, SME, and FRI… However, as all participating laboratories have WGS tools (although not used routinely), it is not likely to have happened, as repeated isolates with reduced susceptibility to carbapenems with no carbapenemase found by their routine diagnostics methodologies would have been submitted to full sequencing, so no outbreak should have been missed during the study. The common slight variations can be allocated to differences in the level of expertise of the users, previous or not experience using MALDI-TOF, or even slight differences in the adjustments of equipments. However, all users accomplished the task without on site training, just with the information provided in this paper, so we proved that this developed methodology is ready to be performed in any microbiology laboratory with minimal equipment and no previous knowledge. As a limitation in our study, we must recognize the low number of clinical isolates in the “clinical validation,” thus concluding that the method is promising, but requires a more robust and comprehensive clinical validation.

The MALDI-TOF hydrolysis assay has the advantage of being able to detect carbapenemase activity regardless of the enzyme produced, including novel enzymes emerging at any given time which are not detected by predefined PCR tools usually used in diagnostics (Yoon et al., 2020). In addition, false positive results can also occur when PCR tools are used, due to the appearance of novel variants which lack carbapenemase activity, such as KPC-28 and OXA-163 (Dortet and Naas, 2017; Oueslati et al., 2019), or with reduced carbapenemase activity, such as OXA-232 and OXA-244 (Potron et al., 2013; Hoyos-Mallecot et al., 2017). MALDI-TOF MS measures carbapenemase activity, acting like a rapid phenotypic method. However, additional techniques are required for precise identification of the carbapenemase class. Recent publications have overcome this issue, demonstrating the potential use of MALDI-TOF MS for carbapenemase classification using different combinations of β-lactamase inhibitors (Carvalhaes et al., 2018; Oviaño et al., 2020). The requirement for testing the isolates from blood agar cultures is a limitation of our study, as no other culture media has been tested. However, in our experience the hydrolysis assay by MALDI-TOF works very similarly using bacterial colonies from other type of culture media as McConkey agar or Mueller-Hinton agar (data not shown). Also, the need of fresh controls could be observed as a limitation of the study, however, most laboratories that have an antimicrobial resistance detection section will have this controls ready to use for different findings.

In comparison with biochemical assays, MALDI-TOF MS has the advantage of overcoming the subjective visual interpretation, particularly in case of weak positive reactions as in OXA-48-like enzymes, where the color change is not as evident. In comparison with other in-house methods, the major advantage of the method is the standardized processing, with no need to establish a particular number of cells of the isolate for analysis and no subsequent extraction (Hrabák et al., 2011; Monteferrante et al., 2016). The excellent performance of the buffer enables an all-in-one (Oviaño et al., 2016), simplified procedure, with sensitivity and specificity values in the same range.

The use of our in-house procedure is more labor intensive than with the MBT STAR^®^-Carba IVD Kit (Bruker Daltonik), as it requires preparation of specific reagents, like the antibiotic solution, matrix and calibration standard, making in principle, the introduction of the method in routine less straightforward. This is why the working protocol with the standardized procedure and educational video for software management is so useful and helps in the familiarization of the methodology (Supplementary Data Sheet 1 and Supplementary Video 1). The different staff performing the assay were not dedicated or expert personnel, but was the same person in each center during the entire study. The preparation of reagents consists in simple steps of weighing and dilution, techniques commonly used in microbiology laboratories that take about 10–20 min to accomplish, with an extra 30 min of incubation. Besides, in the future automated machines can be programmed for preparation of MALDI-TOF reagents in the users way, as there are different protocols for DNA/RNA extraction and for PCR in molecular techniques. Comparing the turnaround time for both techniques, it is quite similar, with a short delay of for the preparation of reagents in our developed in-house procedure, with the additional advantages in the methodology developed herein of a very low cost (∼ 1 euro) and the possibility of adjusting the procedure for any antibiotic (Oviaño et al., 2017).

## Conclusion

MALDI-TOF MS is an outstanding tool for the rapid detection of carbapenemase activity in Enterobacterales in clinical microbiology laboratories. The use of a simple in-house procedure with online software allows results to be obtained within 30–60 min, making MALDI-TOF a rapid phenotypic technique suitable for routine screening of carbapenemases in diagnostics and thus facilitating the early implementation of appropriate antimicrobial therapy.

## Data Availability Statement

The original contributions presented in the study are included in the article/Supplementary Material, further inquiries can be directed to the corresponding author/s.

## Author Contributions

MO: contributions to the conception or design of the work. EG, AA, MArr, MArt, JC, AC, KC, IPC, CE, JF, JJ, PL, GM, XM, and PP-P: contributions to the acquisition and analysis. BR-S, RC, JH, LM, LM-M, AO, ÁP, AV, and GB: contributions to the drafting the work or revising it critically for important intellectual content. GB and MO: provide approval for publication of the content. All authors contributed to the article and approved the submitted version.

## Conflict of Interest

MArr, JJ, GM, and LM were employed by company Clover BioSoft. The remaining authors declare that the research was conducted in the absence of any commercial or financial relationships that could be construed as a potential conflict of interest.

## Publisher’s Note

All claims expressed in this article are solely those of the authors and do not necessarily represent those of their affiliated organizations, or those of the publisher, the editors and the reviewers. Any product that may be evaluated in this article, or claim that may be made by its manufacturer, is not guaranteed or endorsed by the publisher.

## Footnotes

1
https://platform.clovermsdataanalysis.com/login
