# Supplementary material for: Multicenter Performance Evaluation of MALDI-TOF MS for Rapid Detection of Carbapenemase Activity in Enterobacterales: The Future of Networking Data Analysis With Online Software
Source: Front Microbiol. 2022 Jan 27;12:789731. doi: 10.3389/fmicb.2021.789731 (PMC8834885; doi:10.3389/fmicb.2021.789731)
Supplement: Supplementary file 1 [file Data_Sheet_1.PDF]

CLOVER BioSoft

**Clover Bioanalytical Software**
Av. del Conocimiento, 41
18016 Granada (Spain)
info@cloverbiosoft.com
www.cloverbiosoft.com

# Carbapenem Hydrolysis Detection Report

**Exported by:** Marina Oviaño García [marina.oviano.garcia@sergas.es]

**Date:** 2021/04/26 09:41:54 GMT+0200

## Analysis Info

**Project Name:** Multicenter evaluation of the detection of carbapenem hydrolysis by MALDI-TOF MS

**Organisation:** Hospital Universitario de A Coruña

**Run by:** Marina Oviaño García [marina.oviano.garcia@sergas.es]

**Date:** 2021/04/26 09:41:52 GMT+0200

## Analysis Settings

**Antibiotic:** Imipenem - IMI

**Negative Control:** CNEG

**Positive Control:** CPOS

## Analysis Results

*Table result*

| Label Name | RH Norm | Interpretation |
|---|---|---|
| CNEG | -0.1141680484 | Negative |
| CPOS | 1.0077977437 | Positive |
| Sample1 | -0.2991546101 | Negative |
| Sample2 | 1.1129768227 | Positive |
| Sample3 | 1.0735776808 | Positive |

**Clover Bioanalytical Software**
Av. del Conocimiento, 41
18016 Granada (Spain)
info@cloverbiosoft.com
www.cloverbiosoft.com

## *Scatter Plot*

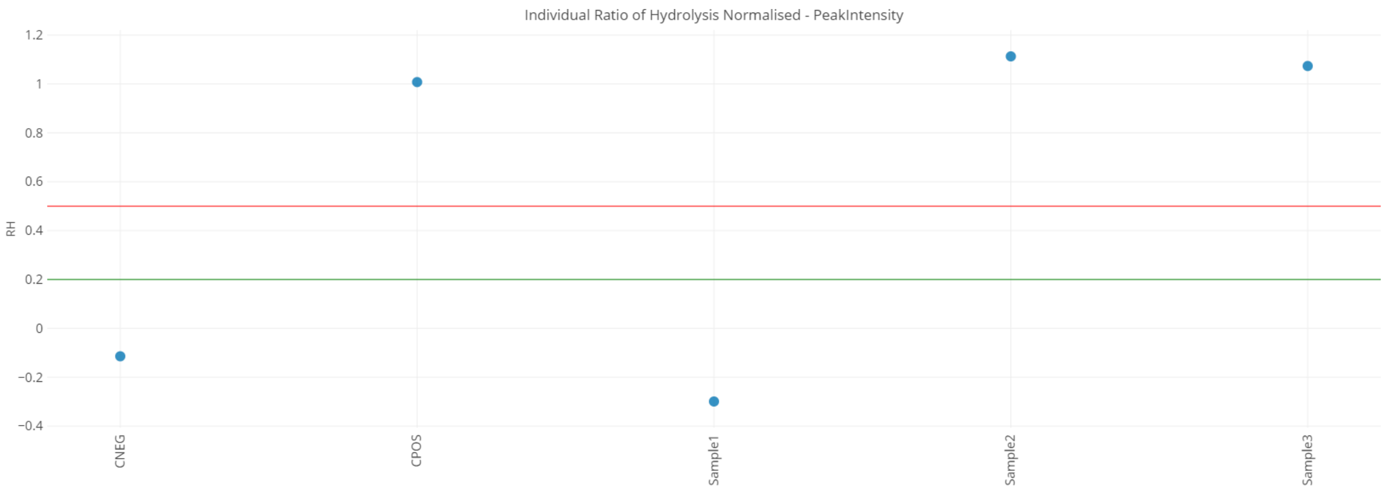


## *Box Plot*

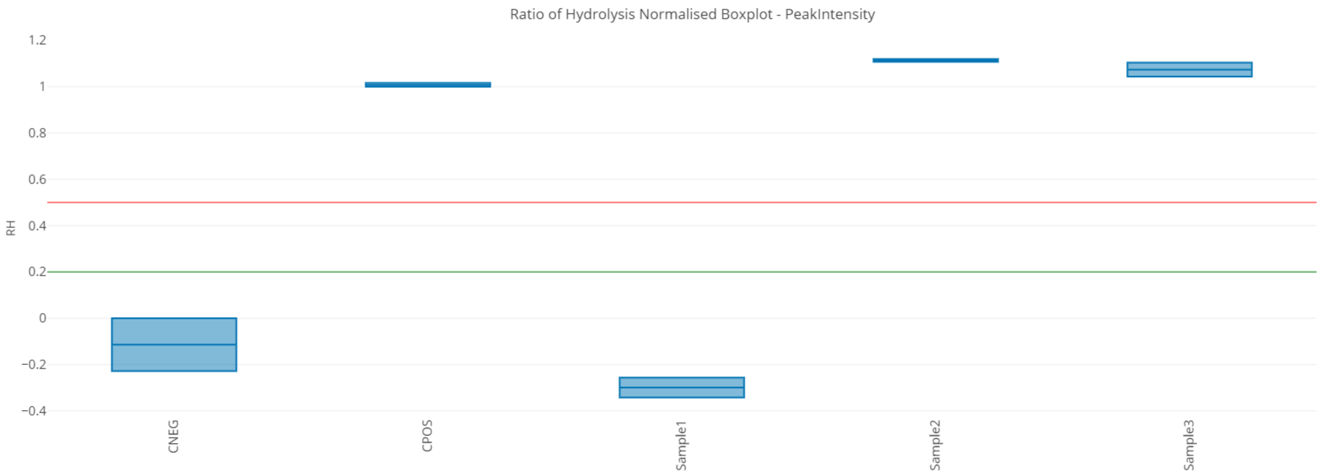

CLOVER BioSoft

**Clover Bioanalytical Software**
Av. del Conocimiento, 41
18016 Granada (Spain)
info@cloverbiosoft.com
www.cloverbiosoft.com

## Conclusions

The beta-lactam hydrolysis assay can only detect a present beta-lactamase and carbapenemase with hydrolytic activity. It is not a susceptibility assay. The RH (ratio of hydrolysis) cannot be related to the minumum inhibitory concentration (MIC) and therefore it is not useful for assigning a clinical susceptibility category. A negative hydrolysis result does not preclude resistance due to other mechaninsms, specially in *Pseudomonas spp* and *Acinetobacter spp.*

A positive value of RH is related to the presence of a carbapenemase enzyme, an intermediate value represents an ambiguos hydrolysis pattern that requires further testing or confirmation by other techniques and a negative value dismisses the posibility of bacteria carrying a carbapenemase enzyme.
