# Supplementary material for: Multicenter Performance Evaluation of MALDI-TOF MS for Rapid Detection of Carbapenemase Activity in Enterobacterales: The Future of Networking Data Analysis With Online Software
Source: Front Microbiol. 2022 Jan 27;12:789731. doi: 10.3389/fmicb.2021.789731 (PMC8834885; doi:10.3389/fmicb.2021.789731)
Supplement: Supplementary file 2 [file Data_Sheet_2.PDF]

# STANDARDIZED OPERATING PROCEDURE FOR DETECTION OF CARBAPENEM HYDROLYSIS BY MALDI-TOF MS

1. **Preparation of the samples**
   a. Use fresh bacterial isolates. (You may analyse as many samples as you want and in any order. We recommend to test batches of 10 isolates, plus the positive and negative control)
   b. The positive and negative control strains should be tested along with the samples in every run.
   c. For each sample, collect 1 – 5 individual bacterial colonies adequately filling 1 µl inoculation loop. Less quantity could lead to a false negative result.

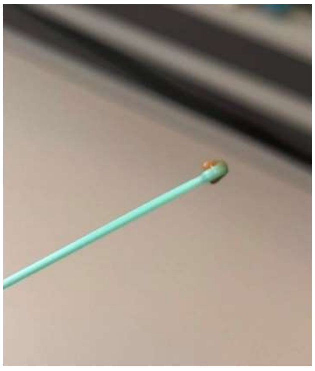

   d. Suspend the bacteria in the loop in the tube containing 50 µl of the antibiotic solution (imipenem 0.5 mg/ml; $NH_4HCO_3$ 10mM + 10µg/ml ZnCl2 + 0,001% SDS, pH=8).
   e. Mix for 5 s using a vortex.
   f. Incubate the sample strains with the positive and negative control strains at 37°C for 30 minutes under slow agitation (≈ 400 rpm).
   g. After incubation, centrifuge the samples (2 min, 14.000 rpm)
   h. Deposit 1.0 µL supernatant onto two different spots in the MALDI target plate.
   i. Deposit 1.0 µL of the calibrator solution onto two different spots in the MALDI target plate.
   j. Allow the spots to dry at room temperature (no artificial light above the target) and overlay each spot with 1.0 µL of the matrix solution (it should be added within 30 min after the samples have dried).
   k. Allow the spots to dry at room temperature and perform the measurement (the target plates should be measured within 2 hours after preparation).

2. **Preparation of the antibiotic solution**

Working buffer

Ammonium bicarbonate 10mM + 10µg/ml $ZnCl_2$ + 0,01% SDS, pH=8

a. **Ammonium bicarbonate**

Stock solution: Dissolve 158 mg of ammonium bicarbonate in 20 ml of water. Shake 10 s in vortex. (Keep it in the fridge for 1 month maximum)

Working solution: Dilute 1 ml of the stock solution in 9 ml of water (quality HPLC). (Keep in the fridge for 15 days maximum).

pH should be 8 without further adjustments, if we need to rise or lower it, we will use an 0.1 N NaOH solution o 10 % of HCl.

**b.** Zinc chloride solution

Dissolve 10 mg of $ZnCl_2$ in 1 ml of water.

Add 10 µl to the working solution.

**c.** Detergent

Weigh 1 mg of SDS y add it to the working solution.

Add 0.5 mg of imipenem in 1 ml of the working buffer.

3. **Preparation of the calibrator**

The calibrator will be a mixture of Bradykinin [1-5] and [1-7] at 35 µM.

1. Mix 1.5 µl of Bradykinin [1-5] (3.5 mM) with 2 µl of Bradykinin [1-7] (2.6 mM).
2. Add 146.5 µl of water.
3. Keep it in the refrigerator (maximum 1 month).

4. **Preparation of the matrix**

Stock solution:

a. Weigh 1 mg of reserpine.

b. Dilute it in 10 ml of organic solvent (acetonitrile 50%, water 47,5% and trifluoroacetic acid 2.5 %).

c. Keep it in the refrigerator (maximum 1 month).

ATB MALDI matrix (1ppm/ µl):

a. 1.5 µl of the stock solution is added to the lyophilized matrix (Matrix HCCA-portioned, Bruker Daltonik).
b. 248.5 µl of organic solvent is added.
c. The matrix is ready to be used. Keep it in a dark box in the refrigerator (maximum 1 week).

5. **Reading**

a. Copy and paste in D:\Methods the methods and mass lists needed for antibiotic spectral acquisition:
   i. FlexControlMethods: MBT_ATB.par
   ii. AutoXMethods: MBT_ATB_AutoX.axe
   iii. MassControlLists: MBT_ATB.mcl
b. Open FlexControl and select the method MBT_ATB.par

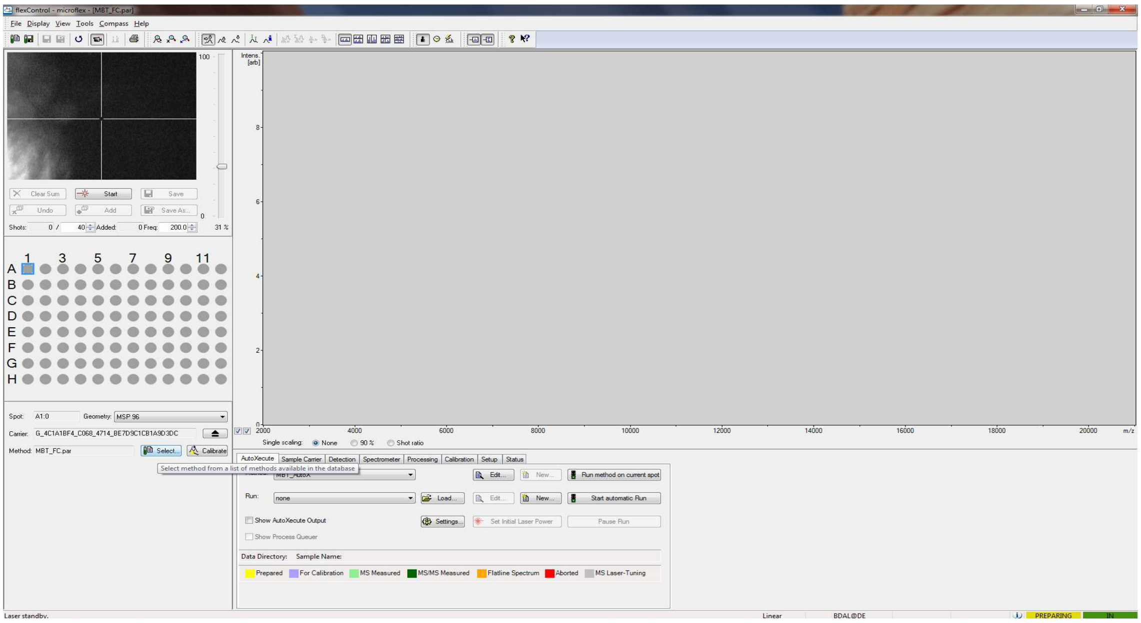

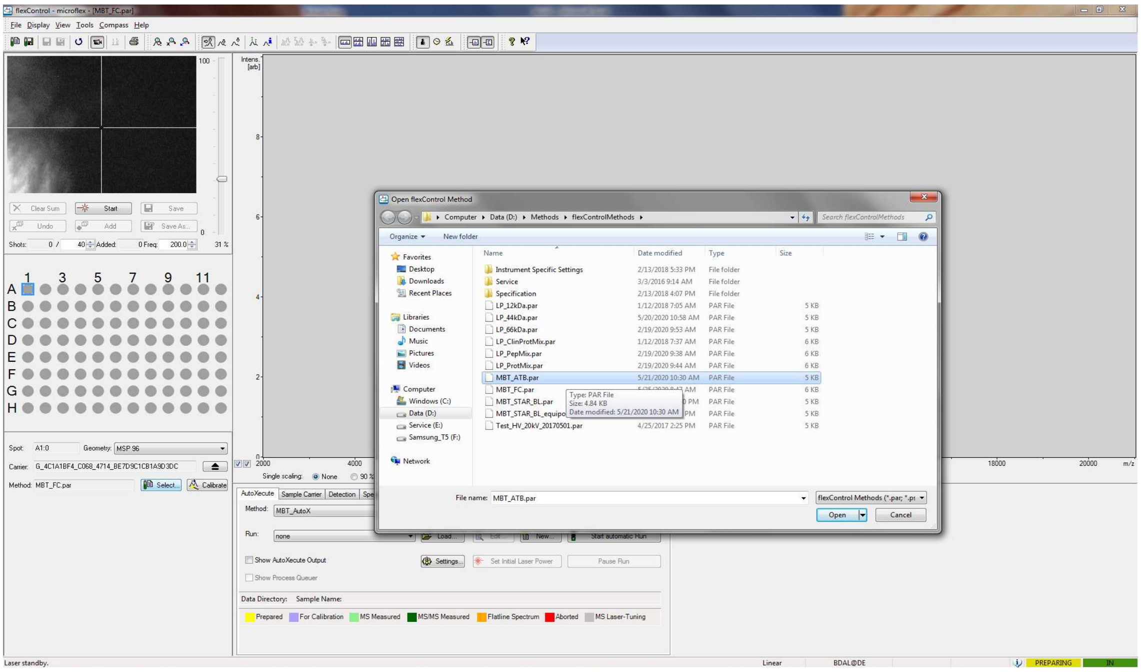

a. For calibration, select the AutoXecute tab, Method: MBT_ATB_AutoX, select the spot for calibration and click on Run Method on Current Spot [there will be two spectra in the window, one adquired after the last shot (display single buffer, top right of the toolbar) and another one that corresponds to the sum of the shots (display sum buffer). Be sure to have selected only the sum buffer if you want calibrate or save the spectra.

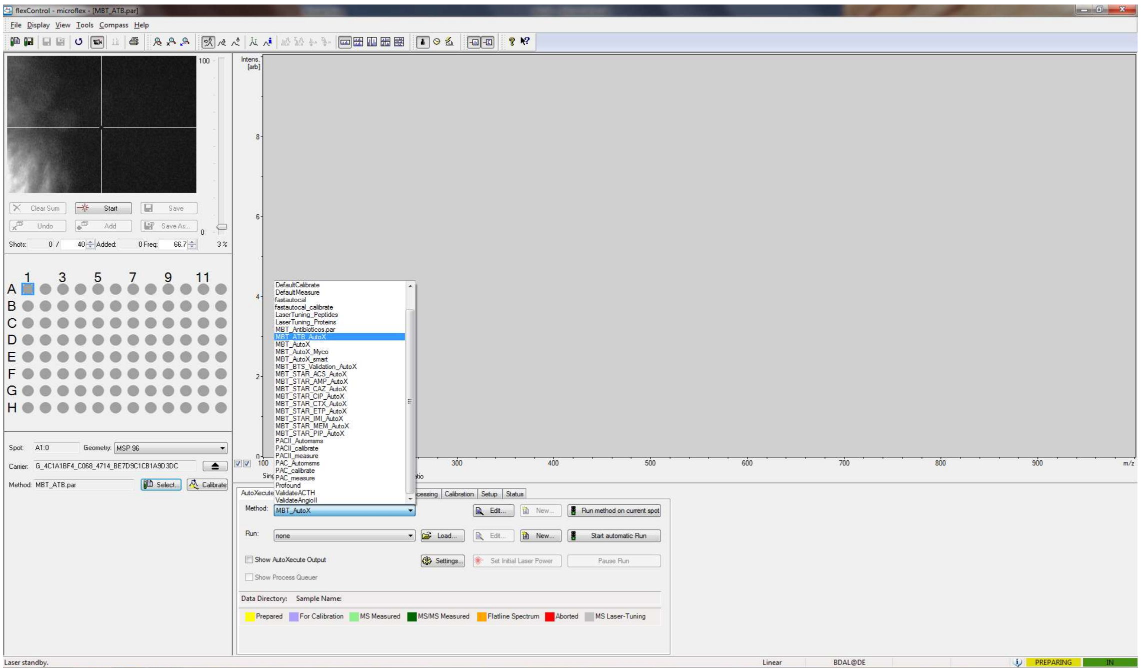

b. Go to the calibration tab. The mass control list should be: MBT_ATB

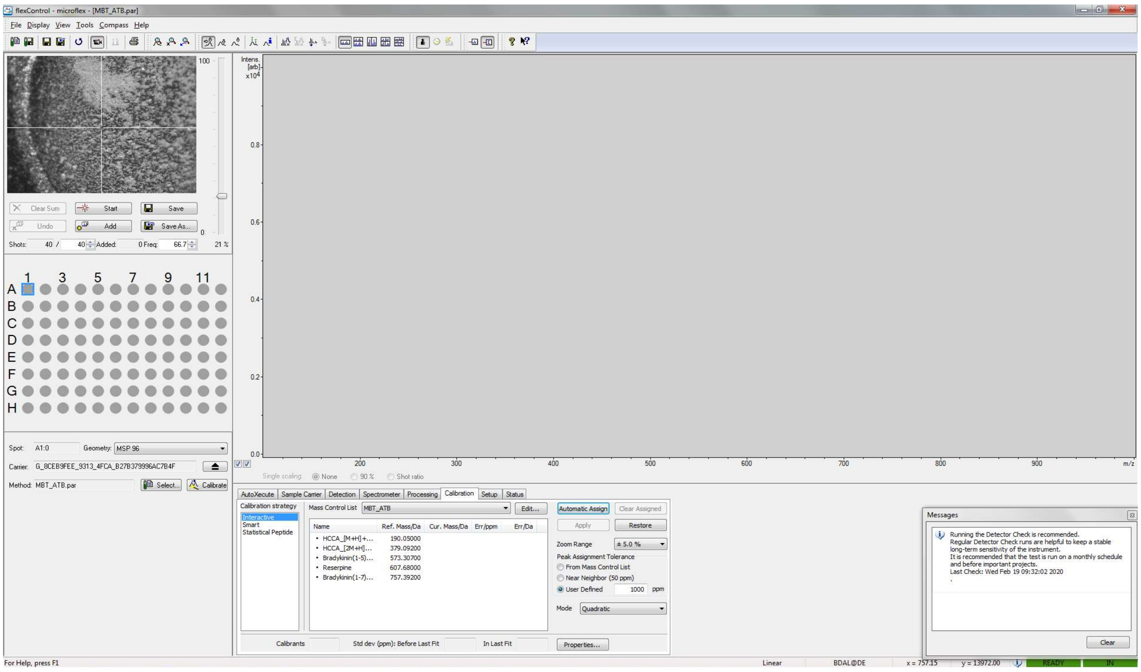

c. Select automatic assign and click apply

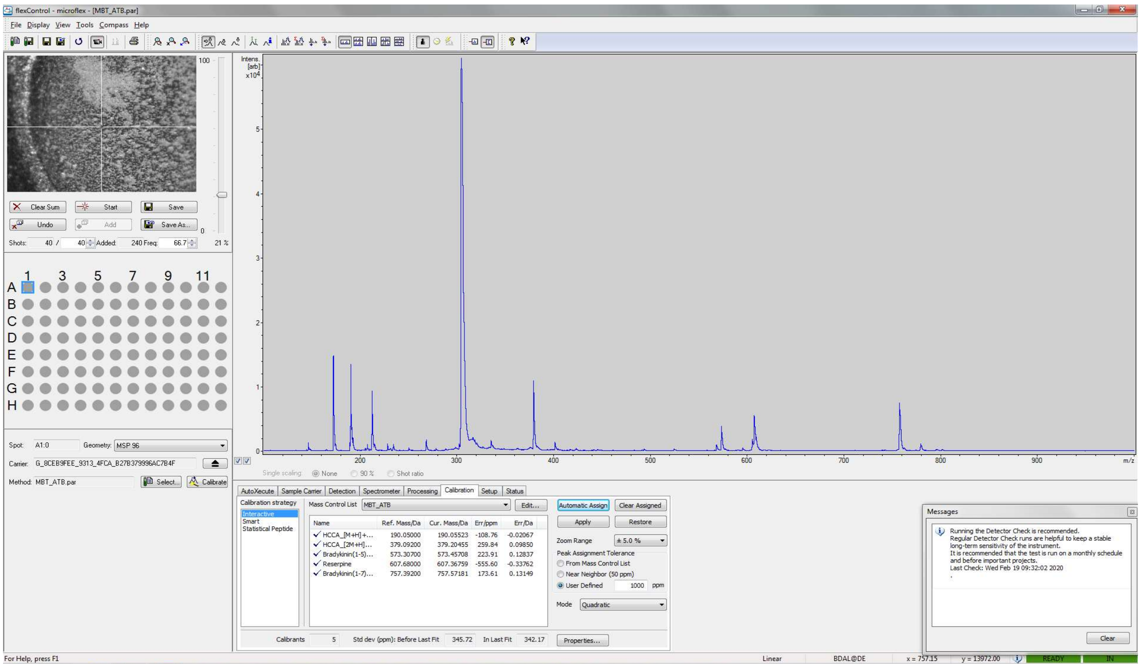

d. If any of the mass peaks is not automatically selected, you can select it in the spectrum manually.

e. The optimum intensities and resolutions for each mass peak should be:

| Name | m/z | Resolution | Intensity |
|---|---|---|---|
| HCCA_$[M+H]^+$ | 190.050 | > 300 | > 3000 |
| HCCA_$[2M+H]^+$ | 379.092 | > 500 | > 3000 |
| Bradykinin (1-5) | 573.314 | > 700 | > 2000 |
| Reserpine | 607.680 | >800 | > 3000 |
| Bradykinin (1-7) | 757.400 | > 1000 | > 3000 |

For checking the values select the mass peaks in the spectra and click Show peak information

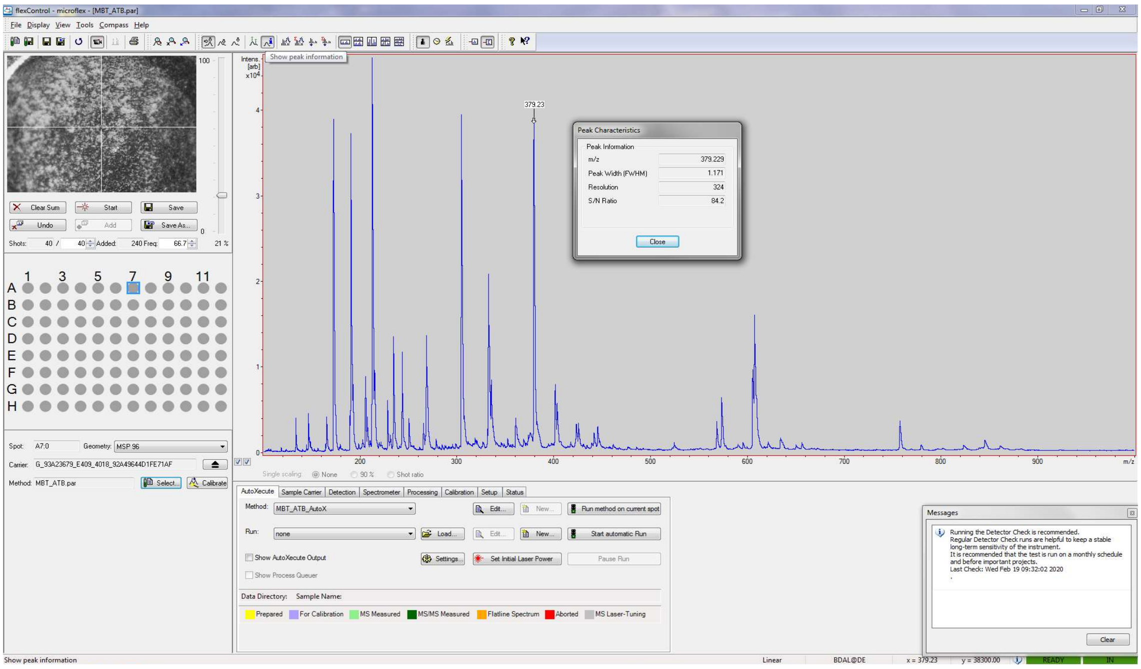


f. For the samples spectral acquisition, select the spot for measuring, then select the AutoXecute tab, Method: MBT_ATB_AutoX and Run Method on Current Spot.

g. When the spectrum acquistion is finished, select display sum buffer (topright of the toolbar) and Save as

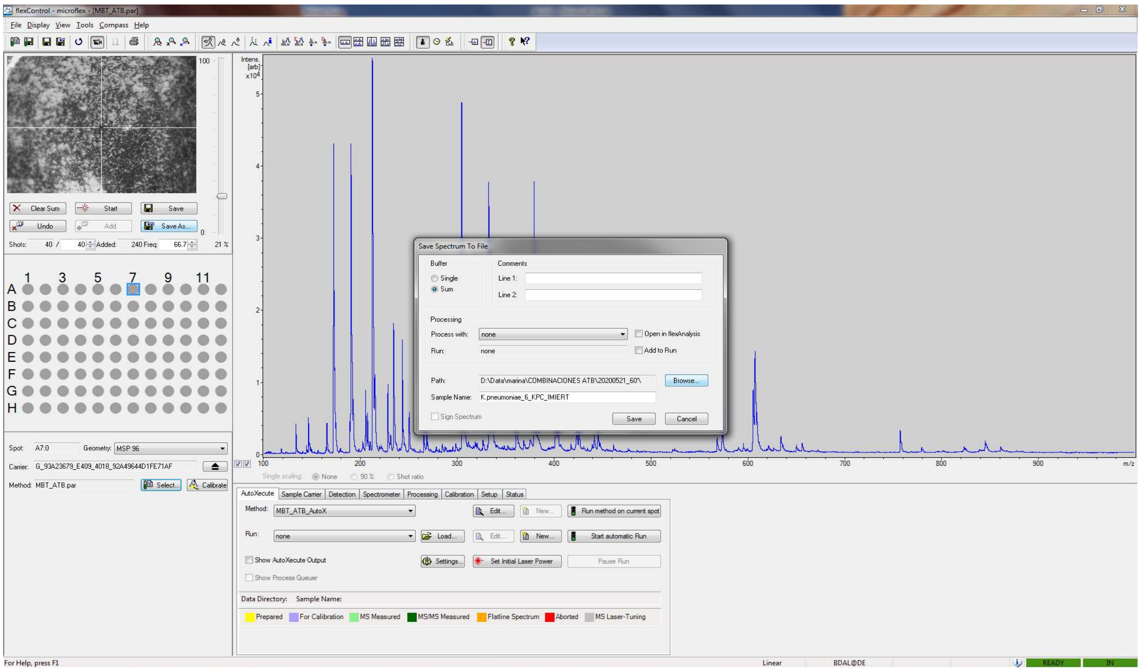


h. The sample name should be the one given by the Complejo Hospitalario A Coruña and the Path should be a general file with the name of each Hospital.

6. **Interpretation**

a. Go to https://platform.clovermsdataanalysis.com/login to connect **'Clover MS Analysis Software'**

b. Log in

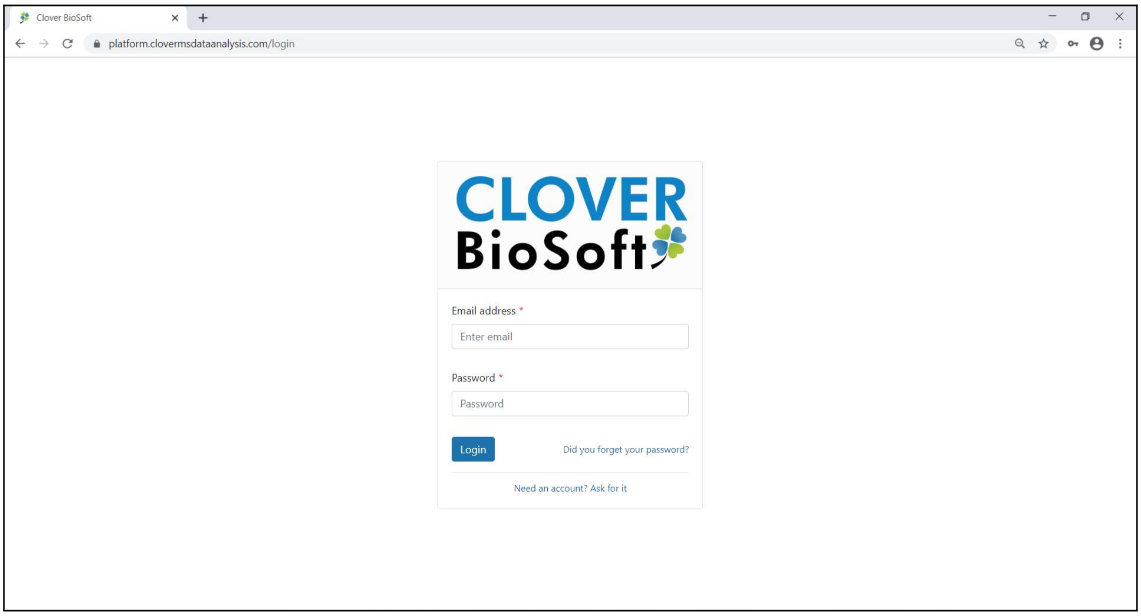


c. Go to 'Analysis' section

d. Select 'Carbapenem Hydrolysis Detection Analysis'

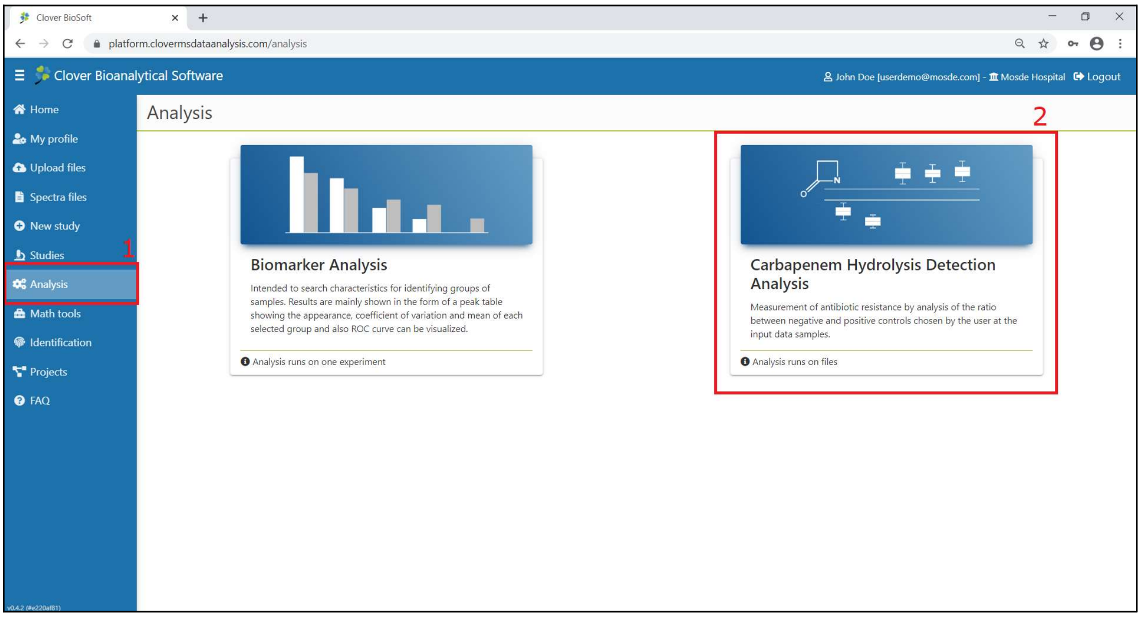


e. Select the project where you want to include the analysis

f. Upload or select files

i. Upload files

1. Choose 'Bruker format'. Your data must be saved in a zip file
2. If first time uploading files, create a new folder called **'Carbapenem Hydrolysis Analysis'** by clicking on 'New folder in root' link
3. Create a subfolder with the date as name by clicking on the folder icon that appears when hovering the 'Carbapenem Hydrolysis Analysis' folder row

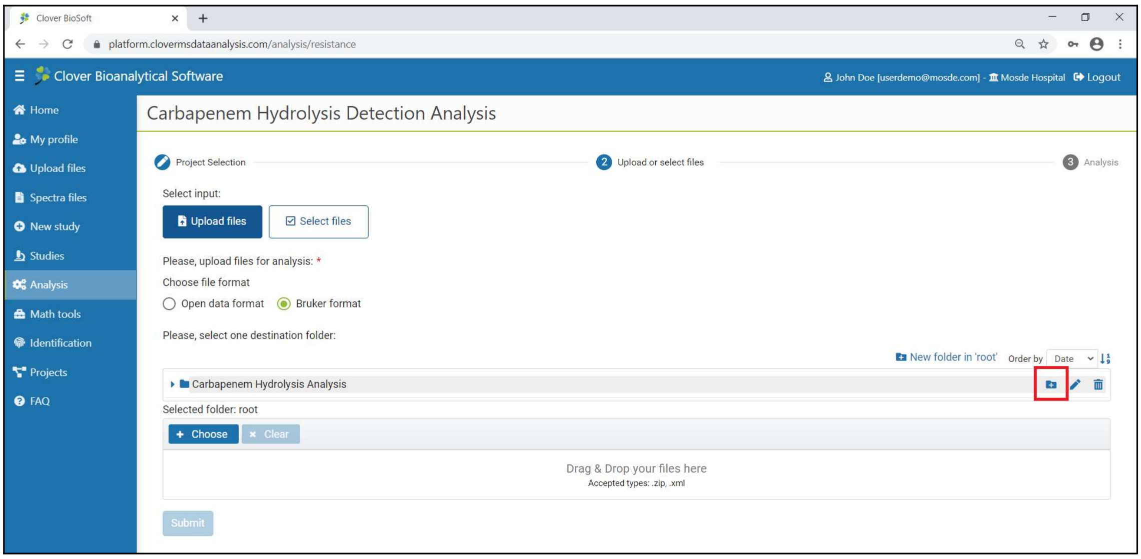

4. Click ‘Choose’ button and then look for and select your files, or add them by ‘Drag and drop’ into the upload area
5. The subfolder must have just the set of files of a unique analysis
6. Click on ‘Submit’ button

ii. Select files

1. Among the folders saved on your data base, search the one with the files you want to analyse
2. Ensure folder is marked with a tick

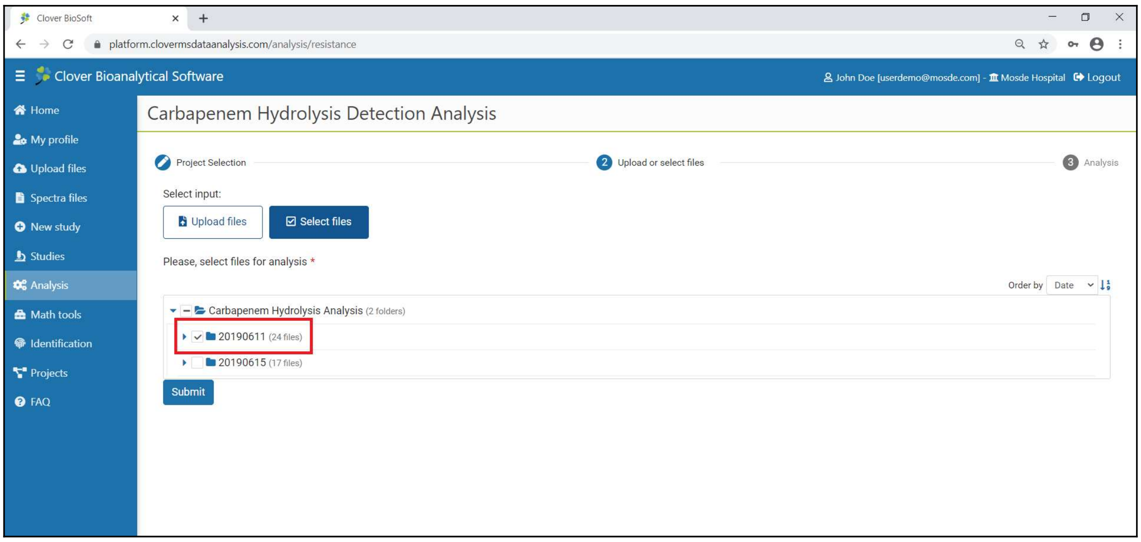


3. Click on ‘Submit’ button

g. Analysis View

i. Select the antibiotic of the study
ii. Choose negative and positive control samples between the ones selected for analysis
iii. Click on ‘Run Analysis’ button

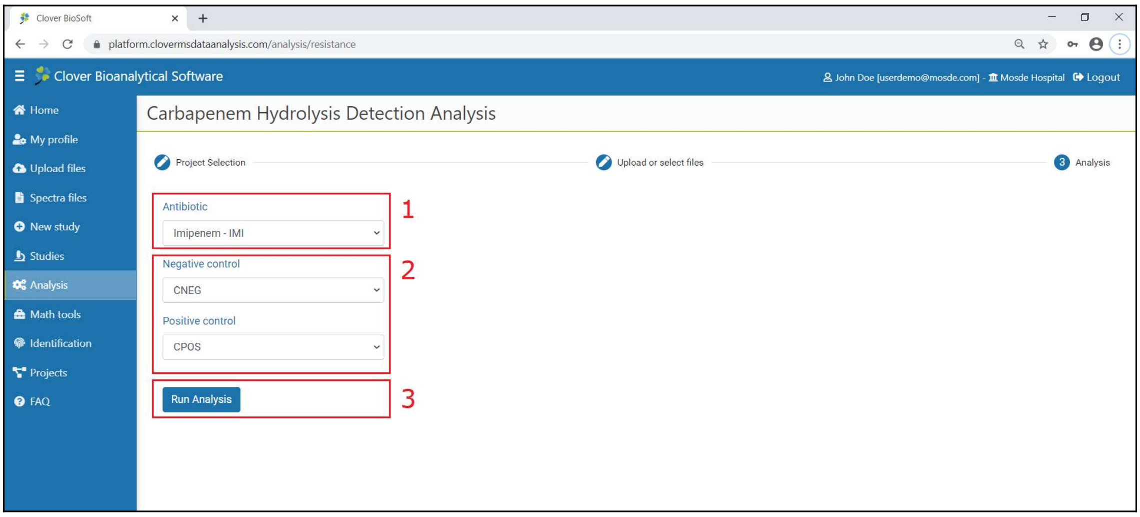


h. Analysis Results

i. Ratio of Hydrolysis Plot. For each sample, the plot shows the ratio of hydrolysis for the selected antibiotic. Values are normalized taking into account the negative and positive controls selected. Horizontal lines for determining the negative and positive region limits are shown

ii. Ratio of Hydrolysis Boxplot. If a sample is composed by two or more replicates, this plot shows the distribution of the set. Data is normalized and regions are also delimited

iii. Spectra plot. A preview of all the samples is available here. Negative and positive controls for the selected antibiotic can be displayed overlapped to the plot

iv. Table result. For each sample, the table shows the ratio of hydrolysis obtained normalizing samples taking into account the samples selected as controls

v. Table boxresult normalized. For each set of samples at the boxplot, the table shows the specific value of the distribution

i. Download PDF report. Click on link and the download will start automatically

j. Save Analysis

i. Click on 'Save analysis' link

ii. A dialogue appears for entering the name of the analysis. A prefix with project name and organization name is automatically added by the system

iii. Click on 'Save' button for saving the analysis

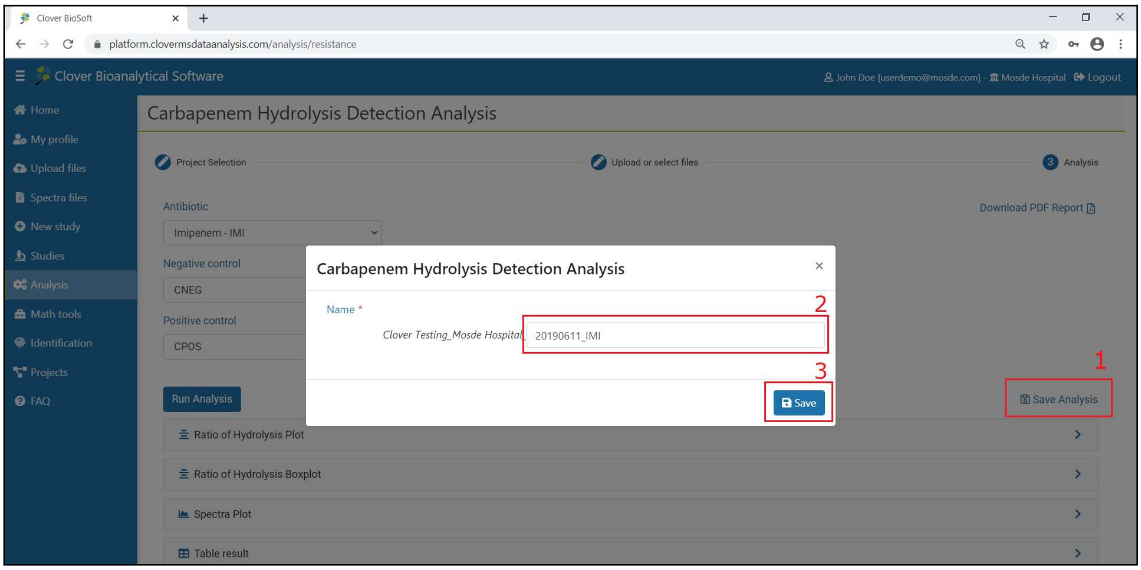


k. View projects and analysis

i. Go to 'Projects' section

ii. Select a project to get information from

iii. Analysis view

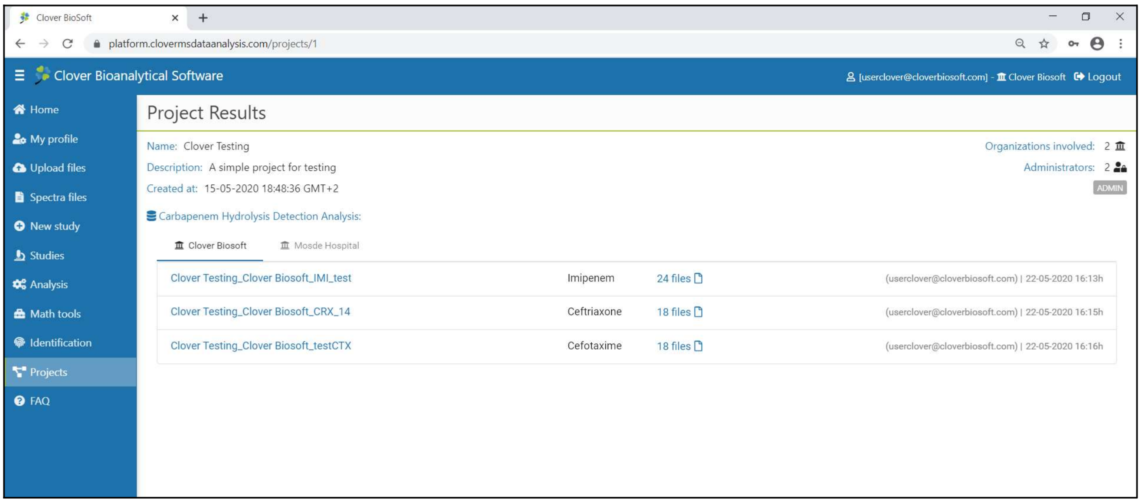


1. You have access to all the results saved by yourself or by any member of your organization
2. In addition, if you are project admin, you have access to all the results linked with the project, whatever organization provided it

3. Select the organization you want to see the results from
    a. Click on the name of any of the analysis
    b. Analysis results are loaded automatically
    c. PDF report can be also generated

7. **Introduction of results in the data base**

   The value of the normalized imipenem ratio of hydrolysis (RHn) should be introduced in the Excel sheet in the column reserved for the corresponding hospital. Once the study has finished, the data should be sent to the PI of the study:

   Marina Oviaño

   Marina.Oviano.Garcia@sergas.es

8. **Technical support:**

   Regarding the laboratory procedure and general attention:

   Marina.Oviano.Garcia@sergas.es

   Regarding software management:

   support@cloverbiosoft.com
