# Supplementary material for: Multicenter Performance Evaluation of MALDI-TOF MS for Rapid Detection of Carbapenemase Activity in Enterobacterales: The Future of Networking Data Analysis With Online Software
Source: Front Microbiol. 2022 Jan 27;12:789731. doi: 10.3389/fmicb.2021.789731 (PMC8834885; doi:10.3389/fmicb.2021.789731)
Supplement: Supplementary file 3 [file Table_1.DOCX]

**Tabla S1.** Characteristic of the isolates included in this study.

| **Isolate** | **Species** | **Carbapenemase production** | **Resistance mechanism** |
| --- | --- | --- | --- |
| 1^1^ | *E. coli* | + | OXA-48 |
| 2^1^ | *E. coli* | + | OXA-48 |
| 3^1^ | *E. cloacae* | + | OXA-48 |
| 4^1^ | *K. pneumoniae* | + | OXA-48 |
| 5^2^ | *K. pneumoniae* | + | KPC-2 |
| 6^2^ | *K. pneumoniae* | + | KPC-2 |
| 7^2^ | *K. pneumoniae* | + | KPC-2 |
| 8^2^ | *E. cloacae* | + | KPC-2 |
| 9^2^ | *K. pneumoniae* | + | KPC-3 |
| 10^2^ | *K. pneumoniae* | + | KPC-2 |
| 11^2^ | *K. pneumoniae* | + | KPC-3 |
| 12^2^ | *K. pneumoniae* | + | KPC-3 |
| 13^2^ | *K. pneumoniae* | + | KPC-3 |
| 14^2^ | *K. pneumoniae* | + | KPC-3 |
| 15^1^ | *E. coli* | + | OXA-48 |
| 16^1^ | *E. coli* | + | OXA-48 |
| 17^1^ | *E. coli* | + | OXA-48 |
| 18^1^ | *E. coli* | + | OXA-48 |
| 19^1^ | *K. pneumoniae* | + | OXA-48 |
| 20^1^ | *K. pneumoniae* | + | OXA-48 |
| 21^3^ | *E. coli* | ±^1^ | OXA-232 |
| 22^3^ | *K. pneumoniae* | ± | OXA-244 |
| 23^3^ | *K. pneumoniae* | + | OXA-245 |
| 24^3^ | *K. pneumoniae* | + | OXA-245 |
| 25^3^ | *K. pneumoniae* | + | OXA-245 |
| 26^3^ | *K. pneumoniae* | + | OXA-245 |
| 27^3^ | *K. pneumoniae* | + | OXA-162 |
| 28^3^ | *K. pneumoniae* | + | OXA-204 |
| 29^1^ | *K. pneumoniae* | + | VIM-1 |
| 30^1^ | *K. pneumoniae* | + | VIM-1 |
| 31^1^ | *E. cloacae* | + | VIM-1 |
| 32^1^ | *K. pneumoniae* | + | VIM-1 |
| 33^1^ | *E. coli* | + | VIM-1 |
| 34^1^ | *E. coli* | + | VIM-1 |
| 35^1^ | *K. pneumoniae* | + | VIM-1 |
| 36^1^ | *E. coli* | + | VIM-1 |
| 37^1^ | *E. coli* | + | VIM-1 |
| 38^1^ | *E. cloacae* | + | VIM-1 |
| 39^1^ | *K. pneumoniae* | + | IMP-22-like |
| 40^1^ | *E. cloacae* | + | IMP-13-like |
| 41^1^ | *E. cloacae* | + | IMP-22-like |
| 42^1^ | *K. pneumoniae* | + | NDM-1 |
| 43^1^ | *K. pneumoniae* | + | NDM-1 |
| 44^1^ | *E. cloacae* | + | NDM-1 |
| 45^1^ | *K. pneumoniae* | + | NDM-7 |
| 46^1^ | *K. pneumoniae* | + | NDM-7 |
| 47^4^ | *K. pneumoniae* | - | CTX-M-14 |
| 48^4^ | *K. pneumoniae* | - | CTX-M-15 |
| 49^4^ | *K. pneumoniae* | - | CTX-M-15 |
| 50^4^ | *K. pneumoniae* | - | CTX-M-15 |
| 51^4^ | *E. cloacae* | - | CTX-M-14 |
| 52^4^ | *K. pneumoniae* | - | CTX-M-15 |
| 53^4^ | *K. pneumoniae* | - | CTX-M-15 |
| 54^4^ | *K. pneumoniae* | - | CTX-M-15 |
| 55^4^ | *E. coli* | - | CMY-2 |
| 56^4^ | *E. coli* | - | CMY-2 |
| 57^4^ | *E. coli* | - | FOX-4 |
| 58^4^ | *E. coli* | - | FOX-4 |
| 59^4^ | *E. coli* | - | FOX-8 |
| 60^4^ | *E. coli* | - | CTX-M-14 |
| 61^4^ | *E. coli* | - | CTX-M-32 |
| 62^4^ | *E. coli* | - | CTX-M-14 |
| 63^4^ | *E. coli* | - | CTX-M-32 |
| 64^4^ | *E. coli* | - | CTX-M-32 |
| 65^4^ | *E. coli* | - | CTX-M-32 |
| 66^4^ | *E. coli* | - | CIT |
| 67^4^ | *K. pneumoniae* | - | SHV |
| 68^4^ | *K. oxytoca* | - | K1 |
| 69^4^ | *E. coli* | - | NC^5^ |
| 70^4^ | *E. coli* | - | NC |
| 71^4^ | *E. coli* | - | NC^5^ |
| 72^4^ | *K. pneumoniae* | - | NC |
| 73^4^ | *K. pneumoniae* | - | NC |
| 74^4^ | *K. pneumoniae* | - | NC |

^1^ These isolates were characterized by PCR and sequencing:

Oteo J, Saez D, Bautista V, Fernández-Romero S, Hernández-Molina JM, Pérez-Vázquez M et al. (2013). Spanish Collaborating Group for the Antibiotic Resistance Surveillance Program. Carbapenemase-producing enterobacteriaceae in Spain in 2012. Antimicrob Agents Chemother. 57: 6344-7.

^2^ Oteo J, Pérez-Vázquez M, Bautista V, Ortega A, Zamarrón P, *et al*. Spanish Antibiotic Resistance Surveillance Program Collaborating Group. (2016). The spread of KPC-producing Enterobacteriaceae in Spain: WGS analysisof the emerging high-risk clones of Klebsiella pneumoniae ST11/KPC-2,ST101/KPC-2 and ST512/KPC-3. J Antimicrob Chemother 71:3392–3399

^3^ Dortet L, Jousset A, Sainte-Rose V, Cuzon G, Naas T. (2016). Prospective evaluation of the OXA-48 K-SeT assay, an immunochromatographic test for the rapid detection of OXA-48-type carbapenemases. J Antimicrob Chemother. 71:1834-40.

^4^ Oviaño M, Gómara M, Barba MJ, Revillo MJ, Barbeyto LP, Bou G. (2017). Towards the early detection of β-lactamase-producing Enterobacteriaceae by MALDI-TOF MS analysis. J Antimicrob Chemother. 72: 2259-2262.

^5^ Carbapenem-reduced susceptibility with no carbapenemase. The MIC are the following: isolate 69, ertapenem= 0,5 mg/ L, imipenem ≤ 0,12 mg/L, meropenem ≤ 0,12 mg/L; isolate 71, ertapenem= 1 mg/ L, imipenem ≤ 0,12 mg/L, meropenem ≤ 0,5 mg/L.
