# Supplementary material for: Multicenter Performance Evaluation of MALDI-TOF MS for Rapid Detection of Carbapenemase Activity in Enterobacterales: The Future of Networking Data Analysis With Online Software
Source: Front Microbiol. 2022 Jan 27;12:789731. doi: 10.3389/fmicb.2021.789731 (PMC8834885; doi:10.3389/fmicb.2021.789731)
Supplement: Supplementary file 4 [file Table_2.DOCX]

**Tabla S*2*. Results of the reference isolates included in this study.**

| **Isolate** | **Species** | **Resistance mechanism** | **Imipenem RH** | | | | | | | | |
| --- | --- | --- | --- | --- | --- | --- | --- | --- | --- | --- | --- |
|  |  |  | **GM** | **RC** | **RS** | **VM** | **SE** | **PZ** | **SL** | **CA** | **AC** |
|  |  |  |  |  |  |  |  |  |  |  |  |
| 1 | *E. coli* | OXA-48 | 1.09 | 1.09 | 1.07 | 0.71 | 0.96 | 0.87 | 1.08 | 0.95 | 1.07 |
| 2 | *E. coli* | OXA-48 | 1.08 | 1.08 | 1.10 | 0.83 | 0.49 | 1.03 | 1.03 | 0.97 | 1.01 |
| 3 | *E. cloacae* | OXA-48 | 1.06 | 1.06 | 0.99 | 0.87 | 1.00 | 1.03 | 1.07 | 1.02 | 1.33 |
| 4 | *K. pneumoniae* | OXA-48 | 1.07 | 1.07 | 1.11 | 0.86 | 0.93 | 0.90 | 1.06 | 1.07 | 1.18 |
| 5 | *K. pneumoniae* | KPC-2 | 1.01 | 1.01 | 1.01 | 0.95 | 1.22 | 1.05 | 0.92 | 0.94 | 0.58 |
| 6 | *K. pneumoniae* | KPC-2 | 1.02 | 1.02 | 1.05 | 0.74 | 0.99 | 1.04 | 1.10 | 0.97 | 0.89 |
| 7 | *K. pneumoniae* | KPC-2 | 1.06 | 1.06 | 1.09 | 0.93 | 0.95 | 1.13 | 0.99 | 1.00 | 0.99 |
| 8 | *E. cloacae* | KPC-2 | 1.10 | 1.10 | 1.01 | 0.84 | 1.03 | 1.08 | 0.96 | 1.16 | 0.82 |
| 9 | *K. pneumoniae* | KPC-3 | 1.10 | 1.10 | 1.08 | 0.82 | 0.78 | 0.96 | 0.90 | 0.93 | 1.40 |
| 10 | *K. pneumoniae* | KPC-2 | 1.08 | 1.01 | 1.04 | 1.10 | 1.12 | 0.92 | 0.57 | 0.94 | 1.42 |
| 11 | *K. pneumoniae* | KPC-3 | 1.03 | 0.83 | 1.09 | 0.93 | 0.96 | 0.97 | -0.02 | 0.81 | 1.43 |
| 12 | *K. pneumoniae* | KPC-3 | 0.92 | 0.84 | 1.10 | 0.61 | 1.05 | 1.05 | 1.30 | 0.83 | 1.62 |
| 13 | *K. pneumoniae* | KPC-3 | 1.06 | 1.06 | 1.19 | 0.83 | 1.10 | 0.94 | 1.28 | 0.83 | 1.31 |
| 14 | *K. pneumoniae* | KPC-3 | 0.89 | 0.73 | 1.13 | 1.02 | 0.95 | 1.11 | 1.21 | 0.72 | 1.08 |
| 15 | *E. coli* | OXA-48 | 0.99 | 0.69 | 1.10 | 1.04 | 0.96 | 1.07 | 0.56 | 0.80 | 1.08 |
| 16 | *E. coli* | OXA-48 | 0.91 | 1.59 | 1.09 | 0.72 | 0.91 | 1.07 | 0.70 | 0.79 | 1.15 |
| 17 | *E. coli* | OXA-48 | 0.97 | 1.82 | 1.08 | 0.69 | 1.07 | 0.99 | 0.97 | 1.00 | 0.64 |
| 18 | *E. coli* | OXA-48 | 0.98 | 1.40 | 1.14 | 0.73 | 1.01 | 1.05 | 1.13 | 0.97 | 1.27 |
| 19 | *K. pneumoniae* | OXA-48 | 0.93 | 1.27 | 1.15 | 0.75 | 1.01 | 0.84 | 1.28 | 1.05 | 1.15 |
| 20 | *K. pneumoniae* | OXA-48 | 0.85 | 1.23 | 1.29 | 0.81 | 0.98 | 0.90 | 1.21 | 1.09 | 1.10 |
| 21 | *K. pneumoniae* | OXA-245 | 1.05 | 1.07 | 1.04 | 1.05 | 0.96 | 0.97 | 1.16 | 0.88 | 1.13 |
| 22 | *K. pneumoniae* | OXA-245 | 0.98 | 0.84 | 1.20 | 0.89 | 0.98 | 1.08 | 0.24 | 0.95 | 1.31 |
| 23 | *K. pneumoniae* | OXA-245 | 1.03 | 0.57 | 1.05 | 0.79 | 1.00 | 1.04 | 1.12 | 1.01 | 1.26 |
| 24 | *K. pneumoniae* | OXA-245 | 1.15 | 1.10 | 0.94 | 0.84 | 1.00 | 0.96 | 1.10 | 0.86 | 0.78 |
| 25 | *K. pneumoniae* | OXA-244 | 0.12 | 0.26 | 1.07 | 0.99 | 0.17 | 0.67 | 0.13 | 0.86 | 0.92 |
| 26 | *K. pneumoniae* | OXA-162 | 1.12 | 1.02 | 1.14 | 0.87 | 0.32 | 1.12 | 0.36 | 0.82 | 1.01 |
| 27 | *K. pneumoniae* | OXA-204 | 0.60 | 0.51 | 1.11 | 0.99 | 1.06 | 0.88 | 1.18 | 0.84 | 0.68 |
| 28 | *E. coli* | OXA-232 | 0.16 | -0.20 | 0.99 | 0.82 | 0.01 | -0.32 | 0.04 | 0.80 | 0.25 |
| 29 | *K. pneumoniae* | VIM-1 | 1.11 | 1.11 | 1.06 | 0.96 | 1.04 | 0.94 | 0.78 | 1.01 | 1.02 |
| 30 | *K. pneumoniae* | VIM-1 | 1.08 | 0.98 | 1.05 | 1.11 | 1.13 | 1.00 | -0.79 | 0.92 | 1.42 |
| 31 | *E. cloacae* | VIM-1 | 0.71 | 0.89 | 1.22 | 0.93 | 1.17 | 0.76 | 0.95 | 1.05 | 1.55 |
| 32 | *K. pneumoniae* | VIM-1 | 0.63 | 0.50 | 0.93 | 1.05 | 1.04 | 0.79 | 0.97 | 0.89 | 1.45 |
| 33 | *E. coli* | VIM-1 | 0.72 | 1.11 | 1.17 | 0.88 | 0.34 | 0.80 | 1.02 | 0.92 | 1.40 |
| 34 | *E. coli* | VIM-1 | 0.21 | 0.81 | 0.25 | 0.99 | 0.20 | 0.89 | 0.86 | 0.03 | 1.41 |
| 35 | *K. pneumoniae* | VIM-1 | 0.98 | 0.47 | 1.09 | 0.88 | 1.20 | 2.97 | 0.86 | 0.89 | 0.89 |
| 36 | *E. coli* | VIM-1 | 1.11 | 0.02 | 0.09 | 1.04 | 0.17 | 0.67 | -0.61 | 0.01 | 1.11 |
| 37 | *E. coli* | VIM-1 | 0.98 | 1.02 | 1.07 | 0.80 | 0.42 | 0.60 | 0.67 | 0.82 | 0.84 |
| 38 | *E. cloacae* | VIM-1 | 0.74 | 1.05 | 1.06 | 0.79 | 1.19 | 1.02 | 0.88 | 0.97 | 1.50 |
| 39 | *K. pneumoniae* | IMP-22-like | 1.07 | 0.94 | 0.94 | 0.96 | 1.09 | 0.91 | 0.58 | 0.87 | 0.96 |
| 40 | *E. cloacae* | IMP-13 like | 1.08 | 0.71 | 1.06 | 0.90 | 1.08 | 0.73 | 0.77 | 0.72 | 0.79 |
| 41 | *E. cloacae* | IMP-22 like | 1.04 | 1.08 | 1.04 | 1.14 | 1.08 | 0.97 | 0.64 | 1.10 | 0.23 |
| 42 | *K. pneumoniae* | NDM-1 | 0.83 | 1.34 | 1.12 | 0.87 | 1.08 | 0.78 | 0.83 | 0.97 | 1.14 |
| 43 | *K.pneumoniae* | NDM-1 | 0.63 | 0.16 | 1.07 | 1.19 | 1.01 | 0.74 | -1.52 | 0.90 | 0.98 |
| 44 | *E. cloacae* | NDM-1 | 0.87 | 0.50 | 1.09 | 0.62 | 1.04 | 0.50 | 1.83 | 0.93 | 1.02 |
| 45 | *K. pneumoniae* | NDM-7 | 1.04 | 1.60 | 0.94 | 0.93 | 0.99 | 1.00 | 0.71 | 0.88 | 1.11 |
| 46 | *K. pneumoniae* | NDM-7 | 1.02 | 1.17 | 0.94 | 0.85 | 1.02 | 0.99 | 0.95 | 0.94 | 1.02 |
| 47 | *K. pneumoniae* | CTX-M-14 | 0.02 | -0.67 | 0.24 | -0.07 | 0.01 | -0.09 | -0.15 | 0.08 | 0.13 |
| 48 | *K. pneumoniae* | CTX-M-15 | 0.06 | 0.18 | 0.10 | 0.07 | 0.01 | -0.01 | -0.08 | 0.16 | 0.19 |
| 49 | *K. pneumoniae* | CTX-M-15 | -0.04 | 0.15 | -0.06 | -0.48 | 0.07 | 0.01 | -0.06 | 0.10 | 0.18 |
| 50 | *K. pneumoniae* | CTX-M-15 | 0.10 | 0.04 | 0.01 | -0.42 | 0.02 | -0.05 | -0.09 | 0.11 | 0.09 |
| 51 | *E. cloacae* | CTX-M-14 | 0.00 | 0.15 | -0.07 | -0.04 | 0.03 | -0.12 | -0.18 | 0.17 | 0.04 |
| 52 | *K. pneumoniae* | CTX-M-15 | -0.06 | -0.27 | 0.05 | 0.09 | 0.01 | -0.30 | -0.11 | 0.20 | 0.18 |
| 53 | *K. pneumoniae* | CTX-M-15 | 0.04 | 0.13 | -0.11 | 0.04 | 0.03 | -0.15 | -0.54 | 0.21 | 0.12 |
| 54 | *K. pneumoniae* | CTX-M-15 | 0.05 | 0.17 | -0.20 | -0.06 | 0.24 | 0.19 | -0.03 | 0.18 | 0.13 |
| 55 | *E. coli* | CMY-2 | 0.09 | 0.09 | -0.08 | -0.16 | 0.01 | -0.18 | -1.31 | 0.29 | 0.06 |
| 56 | *E. coli* | CMY-2 | 0.10 | 0.09 | 0.10 | 0.07 | 0.07 | -0.03 | -1.05 | 0.11 | 0.17 |
| 57 | *E. coli* | FOX-4 | -0.03 | -0.03 | -0.09 | 0.18 | -0.02 | -0.14 | -1.03 | 0.27 | 0.37 |
| 58 | *E. coli* | FOX-4 | 0.05 | 0.01 | -0.09 | 0.05 | 0.04 | -0.22 | -0.09 | 0.08 | 0.03 |
| 59 | *E. coli* | FOX-8 | 0.00 | -0.04 | -0.18 | 0.13 | 0.07 | -0.23 | -0.14 | 0.34 | 0.16 |
| 60 | *E. coli* | CTX-M-14 | 0.08 | 0.05 | -0.13 | 0.09 | 0.08 | -0.05 | -1.29 | 0.24 | 0.06 |
| 61 | *E. coli* | CTX-M-32 | 0.02 | -0.19 | 0.05 | -0.06 | 0.01 | -0.01 | -0.23 | 0.18 | 0.11 |
| 62 | *E. coli* | CTX-M-14 | 0.03 | 0.11 | -0.10 | 0.07 | 0.04 | -0.06 | -0.13 | -0.08 | 0.16 |
| 63 | *E. coli* | CTX-M-32 | -0.15 | 0.00 | -0.89 | -0.38 | 0.01 | -0.15 | -0.01 | -0.06 | 0.22 |
| 64 | *E. coli* | CTX-M-32 | 0.11 | 0.05 | -0.25 | -0.08 | 0.14 | 0.06 | -0.04 | 0.08 | 0.36 |
| 65 | *E. coli* | CTX-M-32 | -0.03 | -0.08 | -0.14 | -0.16 | 0.02 | -0.05 | -0.16 | 0.05 | 0.00 |
| 66 | *E. coli* | CIT | -0.11 | -0.28 | 0.03 | 0.27 | 0.09 | 0.17 | -0.03 | 0.10 | -0.05 |
| 67 | *K. pneumoniae* | SHV | -0.05 | -0.05 | 0.29 | 0.06 | 0.02 | -0.16 | -1.17 | 0.09 | 0.19 |
| 68 | *K. oxytoca* | K1 | 0.02 | 0.14 | -0.10 | 0.08 | -0.01 | -0.34 | -0.05 | 0.03 | 0.04 |
| 69 | *E. coli* | S | 0.06 | -0.07 | -0.12 | 0.16 | 0.13 | 0.09 | -0.03 | 0.07 | 0.14 |
| 70 | *E. coli* | S | 0.06 | -0.03 | -0.07 | -0.25 | 0.08 | -0.26 | -0.06 | -0.01 | 0.03 |
| 71 | *E. coli* | S | 0.01 | -0.09 | -0.11 | 0.00 | 0.06 | -0.02 | -1.05 | 0.10 | 0.28 |
| 72 | *K. pneumoniae* | S | 0.04 | -1.15 | -0.14 | 0.06 | 0.07 | -0.10 | -0.68 | 0.05 | 0.13 |
| 73 | *K. pneumoniae* | S | 0.07 | -0.04 | -0.02 | 0.26 | 0.11 | -0.05 | -0.05 | 0.36 | -0.09 |
| 74 | *K. pneumoniae* | S | 0.00 | 0.00 | 0.17 | 0.16 | 0.12 | -0.23 | -0.14 | 0.20 | 0.02 |
