# Supplementary material for: Multicenter Performance Evaluation of MALDI-TOF MS for Rapid Detection of Carbapenemase Activity in Enterobacterales: The Future of Networking Data Analysis With Online Software
Source: Front Microbiol. 2022 Jan 27;12:789731. doi: 10.3389/fmicb.2021.789731 (PMC8834885; doi:10.3389/fmicb.2021.789731)
Supplement: Supplementary file 5 [file Table_3.DOCX]

**Tabla S3.** Results of the clinical isolates included in this study.

| **Isolate** | **Hospital** | **Species** | **Sample** | **Type of carbapenemase** | **RH** |
| --- | --- | --- | --- | --- | --- |
|  |  |  |  |  |  |
|  |  |  |  |  |  |
| 1 | GM | *E. cloacae* | blood | OXA-48 | 0,13 |
| 2 |  | *K. pneumoniae* | urine | OXA-48 | 0,80 |
| 3 |  | *K. pneumoniae* | urine | OXA-48 | 0,89 |
| 4 |  | *K. pneumoniae* | urine | OXA-48 | 0,05 |
| 5 |  | *K. pneumoniae* | blood | OXA-48 | 0,53 |
| 6 |  | *K. pneumoniae* | urine | OXA-48 | 0,92 |
| 7 |  | *K. pneumoniae* | urine | OXA-48 | 0,86 |
| 8 |  | *K. pneumoniae* | abscess | OXA-48 | 0,75 |
| 9 |  | *K. pneumoniae* | urine | OXA-48 | 0,97 |
| 10 |  | *C. koseri* | urine | OXA-48 | 0,94 |
| 11 |  | *K. pneumoniae* | urine | OXA-48 | 0,95 |
| 12 |  | *K. pneumoniae* | urine | OXA-48 | 1,09 |
| 13 |  | *K. pneumoniae* | wound | OXA-48 | 0,90 |
| 14 |  | *K. pneumoniae* | biopsy | OXA-48 | 0,95 |
| 15 |  | *K. pneumoniae* | urine | OXA-48 | 0,29 |
| 16 |  | *K. pneumoniae* | urine | OXA-48 | 0,89 |
| 17 |  | *K. pneumoniae* | urine | OXA-48 | 0,83 |
| 18 |  | *E. cloacae* | blood | OXA-48 | 0,96 |
| 19 |  | *K. aerogenes* | wound | OXA-48 | 0,89 |
| 20 |  | *K. pneumoniae* | wound | OXA-48 | 1,07 |
| 1 | RC | *K. pneumoniae* | urine | OXA-48 | 0,74 |
| 2 |  | *K. pneumoniae* | catheter | VIM | 1,28 |
| 3 |  | *E. coli* | blood | OXA-48 | 1,04 |
| 4 |  | *E. cloacae* | catheter | VIM | 1,10 |
| 5 |  | *E. cloacae* | urine | KPC | 1,54 |
| 6 |  | *K. pneumoniae* | wound | VIM | 1,21 |
| 7 |  | *E. coli* | abscess | OXA-48 | 1,05 |
| 8 |  | *K. pneumoniae* | urine | KPC | 0,80 |
| 9 |  | *K. pneumoniae* | urine | OXA-48 | 1,09 |
| 10 |  | *K. aerogenes* | blood | OXA-48 | 1,28 |
| 11 |  | *K. pneumoniae* | wound | VIM | 0,90 |
| 12 |  | *E. coli* | urine | OXA-48 | 0,77 |
| 13 |  | *C. freundii* | urine | KPC | 0,95 |
| 14 |  | *C. freundii* | urine | KPC | 1,22 |
| 15 |  | *E. coli* | urine | VIM | 0,68 |
| 16 |  | *K. pneumoniae* | colonization sample | VIM | 0,90 |
| 17 |  | *E. cloacae* | abscess | OXA-48 | 1,26 |
| 18 |  | *E. coli* | colonization sample | VIM | 0,86 |
| 19 |  | *E. coli* | urine | OXA-48 | 1,21 |
| 20 |  | *K. aerogenes* | urine | OXA-48 | 0,34 |
| 1 | RS | *P. mirabilis* | colonization sample | OXA-48 | 0,24 |
| 2 |  | *K. pneumoniae* | urine | KPC | 1,14 |
| 3 |  | *K. pneumoniae* | blood | KPC | 1,04 |
| 4 |  | *E. cloacae* | colonization sample | OXA-48 | 1,02 |
| 5 |  | *P. mirabilis* | urine | OXA-48 | 0,22 |
| 6 |  | *E. coli* | colonization sample | IMP | 1,09 |
| 7 |  | *K. pneumoniae* | colonization sample | IMP | 1,14 |
| 8 |  | *K. oxytoca* | urine | OXA-48 | 1,07 |
| 9 |  | *K. oxytoca* | colonization sample | IMP | 0,93 |
| 10 |  | *E. cloacae* | blood | IMP | 1,10 |
| 11 |  | *K. pneumoniae* | colonization sample | KPC | 1,09 |
| 12 |  | *K. pneumoniae* | urine | KPC | 1,08 |
| 13 |  | *K. pneumoniae* | urine | OXA-48 | 1,10 |
| 14 |  | *K. pneumoniae* | wound | KPC | 1,12 |
| 15 |  | *K. pneumoniae* | urine | KPC | 1,03 |
| 16 |  | *K. pneumoniae* | urine | VIM | 1,11 |
| 17 |  | *K. oxytoca* | colonization sample | VIM | 1,09 |
| 18 |  | *E. coli* | colonization sample | OXA-48 | 1,10 |
| 19 |  | *E. cloacae* | colonization sample | VIM | 1,09 |
| 20 |  | *C. freundii* | colonization sample | VIM | 1,13 |
| 1 | VM | *K. pneumoniae* | blood | KPC-2 | 6,77 |
| 2 |  | *K. pneumoniae* | colonization sample | KPC-3 | 1,84 |
| 3 |  | *K. pneumoniae* | urine | OXA-48 | 2,89 |
| 4 |  | *K. pneumoniae* | colonization sample | IMP-8 | 1,17 |
| 5 |  | *K. pneumoniae* | colonization sample | VIM-1 | 2,51 |
| 6 |  | *K. pneumoniae* | urine | OXA-48 | 3,13 |
| 7 |  | *K. pneumoniae* | urine | OXA-48 | 3,81 |
| 8 |  | *K. pneumoniae* | colonization sample | OXA-48 | 2,01 |
| 9 |  | *K. pneumoniae* | urine | OXA-48 | 6,05 |
| 10 |  | *K. pneumoniae* | abscess | VIM-1 | 3,63 |
| 1 | SE | *E. cloacae* | blood | VIM-1 | 0,19 |
| 2 |  | *E. coli* | urine | OXA-48 | -0,07 |
| 3 |  | *E. coli* | blood | - | 0,05 |
| 4 |  | *E. cloacae* | blood | VIM-1 | 0,68 |
| 5 |  | *E. cloacae* | blood | VIM-1 | 0,82 |
| 6 |  | *E. cloacae* | urine | - | 0,10 |
| 7 |  | *E. cloacae* | colonization sample | VIM-1 | 1,24 |
| 8 |  | *K. pneumoniae* | catheter | - | 0,30 |
| 9 |  | *E. cloacae* | urine | VIM-1 | 1,20 |
| 10 |  | *E. cloacae* | blood | VIM-1 | 1,38 |
| 11 |  | *K. oxytoca* | respiratory sample | VIM-1 | 1,07 |
| 12 |  | *K. oxytoca* | urine | VIM-1 | 1,16 |
| 13 |  | *E. cloacae* | respiratory sample | VIM-1 | 1,19 |
| 14 |  | *K. pneumoniae* | urine | - | 0,12 |
| 15 |  | *K. pneumoniae* | colonization sample | - | 0,02 |
| 16 |  | *E. cloacae* | biopsy | VIM-1 | 1,27 |
| 17 |  | *E. cloacae* | respiratory sample | - | 0,16 |
| 18 |  | *K. pneumoniae* | colonization sample | - | 0,00 |
| 1 | PZ | *P. mirabilis* | respiratory sample | NDM | 0,86 |
| 2 |  | *K. pneumoniae* | blood | NDM | 0,59 |
| 3 |  | *K. pneumoniae* | urine | VIM | 2,52 |
| 4 |  | *E. coli* | colonization sample | OXA-48 | 2,22 |
| 5 |  | *E. coli* | urine | NDM | 3,09 |
| 6 |  | *E. cloacae* | colonization sample | KPC | 3,25 |
| 7 |  | *K. pneumoniae* | colonization sample | NDM | 3,55 |
| 8 |  | *C. freundii* | abscess | NDM | 3,10 |
| 9 |  | *E. coli* | urine | KPC | 2,68 |
| 10 |  | *E. cloacae* | respiratory sample | VIM | 1,46 |
| 11 |  | *E. cloacae* | urine | KPC | 3,18 |
| 12 |  | *C. freundii* | urine | KPC | 3,31 |
| 13 |  | *K. pneumoniae* | urine | OXA-48 | 2,85 |
| 14 |  | *E. coli* | colonization sample | OXA-48 | 0,72 |
| 15 |  | *P. mirabilis* | urine | KPC | 3,40 |
| 16 |  | *K. pneumoniae* | urine | OXA-48 | 3,26 |
| 17 |  | *E. coli* | colonization sample | OXA-48 | 1,10 |
| 18 |  | *E. cloacae* | urine | VIM | 0,97 |
| 19 |  | *M. morganii* | catheter | KPC | 3,14 |
| 20 |  | *C. freundii* | colonization sample | VIM | 1,28 |
| 1 | SL | *K. pneumoniae* | colonization sample | OXA-48 | 0,97 |
| 2 |  | *E. cloacae* | urine | - | -0,11 |
| 3 |  | *K. pneumoniae* | colonization sample | KPC | 0,87 |
| 4 |  | *K. pneumoniae* | colonization sample | NDM | 1,02 |
| 5 |  | *K. pneumoniae* | colonization sample | OXA-48 | 0,20 |
| 6 |  | *K. pneumoniae* | colonization sample | OXA-48 | 1,13 |
| 7 |  | *E. coli* | urine | OXA-48 | 1,38 |
| 8 |  | *S. marcescens* | wound | VIM | 1,18 |
| 9 |  | *E. complex* | colonization sample | NDM | 1,16 |
| 10 |  | *K. pneumoniae* | wound | OXA48 | 1,12 |
| 11 |  | *C. freundii* | colonization sample | NDM | 1,23 |
| 12 |  | *K. pneumoniae* | colonization sample | VIM | 1,37 |
| 13 |  | *E. coli* | colonization sample | OXA-48 | 1,15 |
| 14 |  | *C. freundii* | colonization sample | NDM | 1,16 |
| 15 |  | *E. cloacae* | respiratory sample | NDM | 1,25 |
| 16 |  | *E. cloacae* | blood | NDM | 0,91 |
| 17 |  | *K. pneumoniae* | colonization sample | KPC | 1,03 |
| 18 |  | *E. coli* | colonization sample | NDM | 1,17 |
| 19 |  | *K. pneumoniae* | colonization sample | KPC | 0,95 |
| 20 |  | *C. freundii* | urine | OXA-48 | 1,09 |
| 1 | CA | *K. oxytoca* | colonization sample | VIM | 0,99 |
| 2 |  | *K. pneumoniae* | colonization sample | OXA-48 | 1,00 |
| 3 |  | *K. pneumoniae* | colonization sample | OXA-48 | 1,04 |
| 4 |  | *E. coli* | urine | OXA-48 | 1,08 |
| 5 |  | *E. coli* | colonization sample | OXA-48 | 1,08 |
| 6 |  | *K. pneumoniae* | colonization sample | OXA-48 | 0,98 |
| 7 |  | *K. pneumoniae* | colonization sample | OXA-48 | 1,01 |
| 8 |  | *P. mirabilis* | respiratory sample | - | 0,31 |
| 9 |  | *K. oxytoca* | urine | OXA-48 | 1,02 |
| 10 |  | *E. cloacae* | colonization sample | OXA-48 | 0,93 |
| 11 |  | *E. coli* | blood | OXA-48 | 1,02 |
| 12 |  | *C. freundii* | colonization sample | VIM | 0,92 |
| 13 |  | *K. pneumoniae* | colonization sample | OXA-48 | 1,04 |
| 14 |  | *E. cloacae* | colonization sample | OXA-48 | 0,99 |
| 15 |  | *E. coli* | urine | OXA-48 | 0,95 |
| 16 |  | *K. pneumoniae* | colonization sample | OXA-48 | 0,99 |
| 17 |  | *E. coli* | colonization sample | OXA-48 | 0,98 |
| 19 |  | *K. pneumoniae* | urine | OXA-48 | 0,97 |
| 20 |  | *E. cloacae* | colonization sample | OXA-48 | 0,95 |
